# A semi-supervised Bayesian approach for marker gene trajectory inference from single-cell RNA-seq data

**DOI:** 10.1093/bioinformatics/btaf454

**Published:** 2025-08-13

**Authors:** Junchao Wang, Ling Sun, Nana Wei, Yisheng Huang, Naiqian Zhang

**Affiliations:** School of Mathematics and Statistics, Shandong University at Weihai, Weihai, Shandong 264209, China; School of Mathematics and Statistics, Shandong University at Weihai, Weihai, Shandong 264209, China; Key Laboratory of Carcinogenesis and Translational Research (Ministry of Education/Beijing), Department of Lymphoma, Peking University Cancer Hospital & Institute, Beijing 100142, China; School of Mathematics and Statistics, Shandong University at Weihai, Weihai, Shandong 264209, China; School of Mathematics and Statistics, Shandong University at Weihai, Weihai, Shandong 264209, China

## Abstract

**Motivation:**

Trajectory inference methods are essential for extracting temporal ordering from static single-cell transcriptomic profiles, thus facilitating the accurate delineation of cellular developmental hierarchies and cell-fate transitions. However, numerous existing methods treat trajectory inference as an unsupervised learning task, rendering them susceptible to technical noise and data sparsity, which often lead to unstable reconstructions and ambiguous lineage assignments.

**Results:**

Here, we introduce BayesTraj, a semi-supervised Bayesian framework that incorporates prior knowledge of lineage topology and marker-gene expression to robustly reconstruct differentiation trajectories from scRNA-seq data. BayesTraj models cellular differentiation as a probabilistic mixture of latent lineages and captures marker-gene dynamics through parametric functions. Posterior inference is conducted using Hamiltonian Monte Carlo (HMC), yielding estimates of pseudotime, lineage proportions, and gene activation parameters. Evaluations on both simulated and real datasets with diverse branching structures demonstrate that BayesTraj consistently outperforms state-of-the-art methods in pseudotime inference. In addition, it provides per-cell branch-assignment probabilities, enabling the quantification of differentiation potential using Shannon entropy and the detection of lineage-specific gene expression via Bayesian model comparison.

**Availability and implementation:**

BayesTraj is written in R and available at https://github.com/SDU-W-Zhanglab/BayesTraj and has been archived on Zenodo (DOI: 10.5281/zenodo.16758038).

## 1 Introduction

Single-cell sequencing technologies have revolutionized the study of cellular heterogeneity by enabling transcriptome-wide measurements at single-cell resolution (Shapiro *et al.* 2013, Etzrodt *et al.* 2014, Saelens *et al.* 2019). However, these technologies generate static snapshot data, inherently lacking temporal resolution. This limitation poses a significant challenge for the fine-grained reconstruction of continuous and dynamic cell-state transitions (Kolodziejczyk *et al.* 2015, Bacher and Kendziorski 2016, Weinreb *et al.* 2018). To overcome this challenge, trajectory inference (TI) algorithms have been developed to computationally reconstruct dynamic cellular processes by ordering cells along putative trajectories and assigning pseudotime values that reflect their relative progression within complex biological landscapes (Cannoodt *et al.* 2016, Stassen *et al.* 2021). These approaches have enabled more refined analyses of a broad range of biological processes, including embryonic development (Petropoulos *et al.* 2016), cellular differentiation (Bendall *et al.* 2014), immune cell dynamics (Lönnberg *et al.* 2017), and disease progression (Hanahan and Weinberg 2011).

In recent years, a number of TI methods have been proposed, which generally rely on transcriptomic similarity to infer cellular progression by constructing low-dimensional manifolds or graphs. Early approaches such as Monocle (Trapnell *et al.* 2014, Qiu *et al.* 2017, Cao *et al.* 2019) and Wanderlust (Bendall *et al.* 2014) leveraged graph-based embeddings and diffusion maps to reconstruct developmental trajectories. Subsequent methods, including Slingshot (Street *et al.* 2018) and PAGA (Wolf *et al.* 2019), extended these foundations by clustering cells and fitting principal curves or abstracted graphs to capture lineage structures. More recent approaches, such as Palantir (Setty *et al.* 2019) and VIA (Stassen *et al.* 2021), introduced probabilistic frameworks based on graph-based random walks to model differentiation landscapes and handle complex branching topologies. In parallel, methods like scVelo (Bergen *et al.* 2020) and CellRank (Lange *et al.* 2022) have integrated RNA velocity with similarity measures to incorporate temporal and kinetic information into trajectory inference. Collectively, these methods have greatly advanced our understanding of dynamic cellular processes at single-cell resolution.

Nevertheless, most existing TI methods remain unsupervised, exhibiting several inherent limitations. First, they are highly sensitive to technical noise and data sparsity, distorting the inferred manifold and destabilizing pseudotime ordering. Second, their heavy dependence on hyperparameters such as dimensionality-reduction techniques, clustering resolution, and graph construction heuristics often leads to inconsistent trajectories across experimental replicates or parameter settings. Third, the absence of biologically informed priors increases the risk of producing mathematically coherent yet biologically implausible reconstructions. Semi-supervised TI methods, such as Ouija (Campbell and Yau 2019), address some of these issues by incorporating prior knowledge to constrain the inference, thereby improving robustness, consistency, and interpretability of inferred trajectories. However, such methods are rare and typically limited to linear differentiation trajectories, restricting their capacity to capture complex, branching developmental programs.

To fill this gap, we have developed BayesTraj, a novel semi-supervised trajectory inference framework that integrates biologically informed priors, including known lineage topology and marker-gene expression patterns, into a hierarchical generative Bayesian model. BayesTraj simultaneously infers pseudotime, lineage proportions, and marker-gene dynamic parameters, while providing per-cell branch-assignment probabilities. Through extensive benchmarking on both simulated and real datasets, BayesTraj consistently outperforms state-of-the-art methods in pseudotime estimation. Furthermore, it quantifies cellular differentiation potential (DP) and employs Bayesian model comparison to identify branch-dependent genes. By incorporating prior biological knowledge, BayesTraj provides a rigorous and interpretable approach for dissecting complex cell differentiation processes.

## 2 Materials and methods

### 2.1 Data resources

We evaluated BayesTraj on both simulated and real single-cell RNA-seq (scRNA-seq) datasets. Simulated datasets were generated by numerically solving stochastic differential equations (SDEs) that model gene expression dynamics (Chu *et al.* 2016). The simulations were designed to emulate a differentiation process with a predefined trifurcating topology, governed by a synthetic gene regulatory network. Each lineage exhibited distinct activation patterns of marker genes, enabling rigorous evaluation of BayesTraj’s ability to recover pseudotemporal progression, branch structure, and gene activation dynamics. Real datasets were obtained from publicly available scRNA-seq datasets in the GEO and ENA repository: GSE75748 (Chu *et al.* 2016) and GSE98664 (Hayashi *et al.* 2018) for directed differentiation; GSE52583 (Treutlein *et al.* 2014) and PRJEB23303 (Jia *et al.* 2018) for bifurcating trajectories; GSE70245 (Olsson *et al.* 2016) for a trifurcating lineage (Table 1, available as supplementary data at *Bioinformatics* online). Marker genes were either user-specified or extracted from dataset metadata (Table 2, available as supplementary data at *Bioinformatics* online). For robust inference, we recommend at least four marker genes per lineage. Details of simulated data generation and real data preprocessing are provided in the Supplementary Materials, available as supplementary data at *Bioinformatics* online.

### 2.2 The Bayesian mixture model

We begin with an N×G gene expression matrix, where N denotes the number of cells and G the number of selected marker genes. The row vector $y_{i}$ represents the expression profile of cell i. For each cell, we define a latent continuous variable $t_{i}\in [0,1]$ representing its pseudotime, and a latent discrete variable $z_{i}\in \{1,\;2,\ldots ,K\}$ indicating the differentiation trajectory to which it belongs. Conditional on $z_{i}=k$, the observed expression $y_{i}$ is assumed to follow a multivariate normal distribution:

$${\;y_{i}∼N(\mu }_{i}(t_{i},\Theta^{k}),{\;\Sigma }_{i}(t_{i},\Theta^{k})),$$

where $\Theta^{k}\;$denotes the set of parameters characterizing the gene expression dynamics for trajectory k.

Both the mean $\mu_{i}(t_{i},\Theta^{k})$ and the variance $\Sigma_{i}(t_{i},\Theta^{k})$ are explicitly modeled as time-dependent functions. For genes that serve as markers of trajectory k, we adopt a switch-like logistic function:

$${\;\mu }_{ij}(t_{i},\Theta_{j}^{k})=\frac{2\delta_{j}}{1+\text{exp}\;(-\tau_{j}((t_{i}-t_{j}^{\left(0\right)}))\;},$$

in which $\tau_{j}$ represents the activation steepness, $t_{j}^{0}$ denotes the activation time, and $\delta_{j}$ controls the maximal amplitude. For non-marker genes, we instead employ a transient Gaussian pulse:

$$\mu_{ij}(t_{i},\Theta_{j}^{k})=2\eta_{j}\;\text{exp}\;{\left(-\zeta_{j}(t_{i}-t_{j}^{\left(0\right)}\right)}^{2}),$$

where $\zeta_{j}$ controls the width of the pulse, $t_{j}^{0}$specifies the pulse midpoint, and $\eta_{j}$ represents its peak magnitude. We assign normal priors on the amplitude and shape parameters ($\delta_{j}$, $\tau_{j}$, $\eta_{j}$, $\zeta_{j}$), and a Beta prior on the temporal location $t_{j}^{\left(0\right)}$. These priors can either be specified based on biological knowledge or inferred from the data in an empirical Bayes framework.

To model gene-specific overdispersion, the variance is parameterized as a function of the mean:

$${\;\Sigma }_{ij}(t_{i})=(1+\phi ){\;\mu }_{ij}(t_{i},\Theta_{j}^{k})+\epsilon ,$$

where ϕ∼Gamma(·) is a gene-specific dispersion parameter and ϵ=0.01 is a small constant added for numerical stability.

To account for zero inflation arising from technical dropouts in scRNA-seq data, we introduce a binary latent variable $\gamma_{ij}\in \{0,\;1\}$, indicating whether gene j in cell i is subject to dropout. The dropout probability depends on the expected expression level:

$$\gamma_{ij}∼\mathrm{Bernoulli}\;({\mathrm{logit}}^{-1}(\beta_{0}+\beta_{1}{\;\mu }_{ij}(t_{i},\Theta_{j}^{k}))),$$

where $\beta_{0}$, $\beta_{1}∼\;N(0,\;0.01)$ are global dropout parameters.

The overall generative process follows a hierarchical structure:

$$\begin{array}{c} t_{i}∼\mathrm{Uniform}\;(0,\;1),\\ \begin{array}{c} {\pi_{1},\pi_{2},\ldots ,\pi }_{K}\;∼\;\mathrm{Dirichlet}\;(1/K,\ldots ,1/K),\\ \begin{array}{c} z_{i}∼\mathrm{Categorical}\;({\pi_{1},\pi_{2},\ldots ,\pi }_{K}),\\ \begin{array}{c} p_{k}(y_{ij}|{\;\mu }_{ij}(t_{i},\Theta_{j}^{k}),\;{\;\Sigma }_{ij}(t_{i},\Theta_{j}^{k}))\\ =\gamma_{ij}\cdot I(y_{ij}=0)+(1-\gamma_{ij})\cdot N({\;\mu }_{ij}(t_{i},\Theta_{j}^{k}),{\;\Sigma }_{ij}(t_{i},\Theta_{j}^{k})). \end{array} \end{array} \end{array} \end{array}$$

The conditional likelihood of observing the expression vector $y_{i}$ is given by marginalizing over the latent trajectory assignment:

$$p(y_{i}|t_{i},\pi ,\Theta )=\sum_{k=1}^{K}\pi_{k}\cdot \prod_{j=1}^{G}p_{k}(y_{ij}|{\;\mu }_{ij}(t_{i},\Theta_{j}^{k}),\;{\;\Sigma }_{ij}(t_{i},\Theta_{j}^{k})),$$

where ${\pi_{1},\pi_{2},\ldots ,\pi }_{K}$ are lineage proportions, representing the relative occupancy of each differentiation trajectory.

Finally, under the assumption of conditional independence across cells, the joint likelihood of the entire dataset Y, represented as a matrix of marker-gene expression for N cells, is given by:

p(Y|T,π,Θ)=∏i=1Np(yi|ti,π,Θ).

### 2.3 Inference and implementation

To facilitate stable convergence of the generative model, we apply gene-wise min–max normalization on the log-transformed expression matrix, independently rescaling each gene’s expression profile to the [0,1] interval. Bayesian inference of the joint posterior is then performed as follows:

p(T,π,Θ|Y)∝p(Y|T,π,Θ)p(T)p(π)p(Θ),

using Hamiltonian Monte Carlo (HMC) with the No-U-Turn Sampler (NUTS), implemented in Stan via the *rstan* R package. We run four independent chains, each with 6000 iterations (including 3000 warm-up), initialized from prior distributions. Convergence is assessed using $\hat{R}<1.05$ and effective sample sizes exceeding 200. Posterior samples after warm-up are pooled to estimate marginal posterior distributions, with parameters summarized by their posterior modes.

Branch-assignment probabilities are computed in the Stan generated quantities block. For cell i, the probability of assignment to each branch k is given by:

p(zi=k|yi,ti,  μi, Σi,π)=πk · pk(yi| μi, Σi)∑k=1Kπk· pk(yi| μi, Σi) . 

After pooling all NUTS draws, each cell’s final branch-assignment probabilities are summarized by the posterior mode across all samples, denoted $\hat{p}(z_{i}=k)$. We define:

$${\hat{p}}_{\max,i}=\underset{k=1,\ldots ,K}{\max}\hat{p}(z_{i}=k),$$

and apply a user-specified threshold τ. If ${\hat{p}}_{\max,i}\geq \tau$, cell i is assigned to the terminal branch ${k_{i}}^{*}=\underset{k}{\mathrm{argmax}}\hat{p}(z_{i}=k)$; otherwise, it is classified as a progenitor cell. This framework assigns each branch a heterogeneous mixture of both committed and multipotent progenitor cells, thereby enabling robust discrete lineage labeling for downstream analyses.

### 2.4 Computational complexity and runtime

The computational complexity of BayesTraj is primarily determined by HMC-based posterior sampling. Empirically, the runtime scales approximately as O(N×G×K), where N is the number of cells, G is the number of marker genes, and K is the number of branches. On a system equipped with an Intel Xeon 2.10 GHz processor and 16 GB of RAM, inference on a dataset comprising 100 cells typically completes within a few minutes, whereas analysis of 500 cells requires approximately 45 min. Accordingly, datasets containing on the order of a few hundred cells, 10–20 marker genes, and one to three branches generally attain convergence within one hour under default sampling parameters.

### 2.5 Trajectory inference performance evaluation

Since BayesTraj incorporates the ground-truth branch topology as a prior, guaranteeing perfect reconstruction of the developmental backbone, our evaluation focused on pseudotime ordering accuracy. We compared BayesTraj against 11 state-of-the-art TI methods (Table 3, available as supplementary data at *Bioinformatics* online) on simulated and real datasets. For directed differentiation datasets, we also included Ouija, a generative model originally developed for linear differentiation, as BayesTraj recapitulated Ouija’s framework in the single-branch setting but generalized to more complex multi-branch lineages.

On the simulated dataset, we quantified pseudotime accuracy using three correlation metrics between the inferred pseudotime $\hat{T}$ and the ground-truth time T: Pearson correlation r, Spearman correlation ρ, and Kendall’s tau correlation τ, defined as:

r=∑i=1n(t^i-t^¯)(ti-t¯)∑i=1n(t^i-t^¯)2∑i=1n(ti-t¯)2,ρ=cov(rgt^,rgt)σrgt^ σrgt,τ=1n(n−1)∑i≠jsgn(t^i-t^j)sgn(ti-tj),

where $rg_{\hat{t}}$ and $rg_{t}\;$are the ranks of cells, with covariance $\mathrm{cov}(rg_{\hat{t}},rg_{t})$ and standard deviations $\sigma_{rg\hat{t}}$ and $\sigma_{\mathrm{rgt}}$.

For real datasets lacking ground-truth time, we assessed branch assignments using the ${\;F}_{1}$ score. Each terminal lineage (i.e. terminal fate) was treated as a binary classification task. For a given terminal lineage b, TP is the number of cells correctly predicted to belong to b, FP is the number of cells incorrectly assigned to b, and FN is the number of cells belonging to b but assigned elsewhere. Precision and recall for branch b are then computed as:

Precision=TPTP+FP, Recall=TPTP+FN,F1=2×Precision×RecallPrecision+Recall.

We further validated the inferred pseudotime by correlating it with expression profiles of marker genes withheld during model fitting, using Pearson, Spearman, and Kendall metrics to assess temporal concordance.

### 2.6 Differentiation potential estimation and assessment

Among the methods compared, only BayesTraj, Palantir, and VIA provide per-cell branch-assignment probabilities. Accordingly, we quantify each cell’s differentiation potential (DP) using the Shannon entropy of its branch probabilities:

DPi=-∑k=1Kpi,klog⁡pi,k,

where $p_{i,k}$ represents the probability that cell i is assigned to branch k, and K is the total number of branches. Higher entropy reflects greater uncertainty in lineage commitment and increased cell-fate plasticity.

To evaluate the biological relevance, we employed two strategies. First, for the simulated dataset with known ground-truth time, we computed the Spearman correlation between estimated potential and ground-truth time, expecting a negative correlation as cells committed. Second, for real data with discrete cell-state labels, we conducted a Kruskal–Wallis (KW) test to assess global differences in potential across states, followed by one-tailed Mann–Whitney U (MWU) tests between adjacent states to identify significant transitions. Notably, for two-group comparisons, the MWU test is equivalent to the KW test.

### 2.7 Branch-specific gene expression analysis

Branch-specific genes are identified through Bayesian model comparison between two competing hypotheses. The null model ($M_{0}$) assumes constant expression, representing non-dynamic behavior:

$$y∼N(\mu ,\sigma^{2}).$$

In contrast, the alternative model ($M_{1}$) captures dynamic activation using a sigmoidal function:

$$y∼N(\frac{2\delta }{1+\exp(-\tau ((t-t^{\left(0\right)}))\;},\;\sigma^{2}),$$

where δ denotes the maximum expression amplitude, τ the activation steepness, and $t^{\left(0\right)}\;$the activation onset time. All parameters are inferred using Hamiltonian Monte Carlo (HMC) sampling.

We compared the two models using the Bayes factor (${BF}_{10}$), which quantifies the relative evidence in favor of the alternative model ($M_{1}$) over the null model ($M_{0}$):

$${\;BF}_{10}=\frac{p(\mathrm{data}|M_{1})}{p(\mathrm{data}|M_{0})},\;$$

where p(data|M) is the marginal likelihood of the data under model M. Based on the Jeffreys-Kass and Raftery scale, a Bayes factor ${BF}_{10}>10\;$indicates strong evidence for alternative model, ${1<BF}_{10}\leq 10$ suggests moderate evidence, and ${BF}_{10}\leq 1$ supports the null hypothesis.

## 3 Results

### 3.1 Overview of BayesTraj

**Figure 1.**
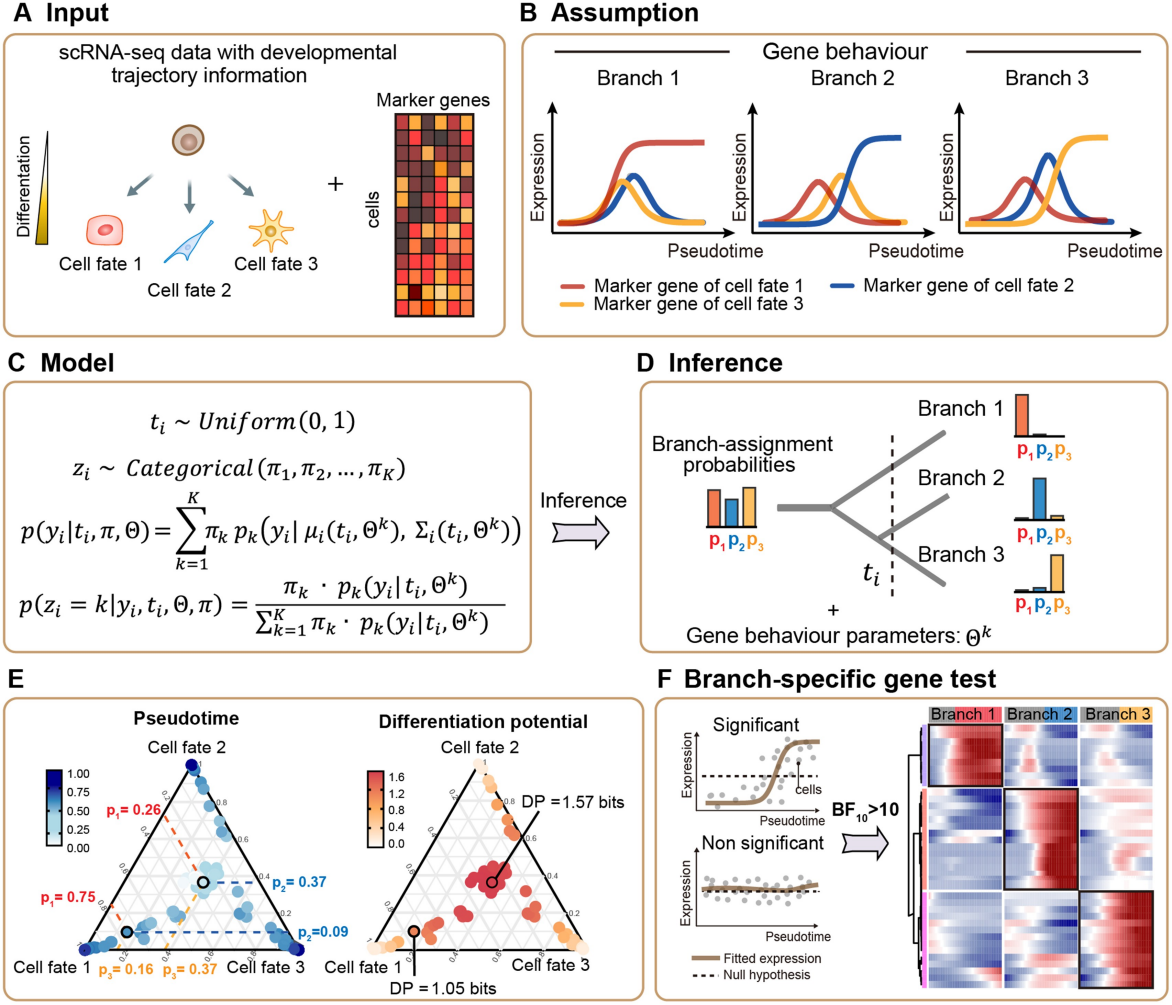
Overview of BayesTraj. (A) BayesTraj takes as input a predefined branching topology and marker-gene expression profiles. (B) Modeled marker-gene behaviors across three branches: switch-like activation in relevant lineages and transient expression otherwise. (C) Hierarchical generative model of BayesTraj. (D) Bayesian inference using HMC yields the joint posterior distribution of pseudotime, lineage proportions, and dynamic parameters for marker genes, along with per‐cell branch-assignment probabilities. (E) Visualization of cell branch‐assignment in the probability simplex, colored by predicted pseudotime (left) and estimated differentiation potential (right). (F) Branch-specific gene testing identifies genes exhibiting significant time-related expression changes.

Assuming that a limited panel of marker genes encapsulates essential regulatory transitions, their expression dynamics provide a principled reference for inferring each cell’s position along a continuous differentiation trajectory. We developed BayesTraj, a semi-supervised trajectory inference framework built upon a hierarchical Bayesian mixture model (Fig. 1). In contrast to conventional unsupervised methods, which typically utilize marker genes only for post-hoc validation, BayesTraj explicitly incorporates marker-gene expression dynamics as priors in its generative model, thereby improving pseudotime accuracy and yielding biologically interpretable trajectories.

BayesTraj takes as input a predefined branch topology and a marker-gene expression matrix (Fig. 1A). We model differentiation as a mixture of latent developmental lineages, each governed by branch-specific parametric models describing marker-gene expression dynamics (Fig. 1B). By integrating these lineage models into a unified probabilistic mixture framework, BayesTraj captures the entire developmental manifold (Fig. 1C). All model parameters, including pseudotime, lineage proportions, and dynamic parameters of marker genes, are jointly inferred via HMC, yielding an approximate posterior over the set of latent variables (Fig. 1D).

In addition to pseudotime inference, BayesTraj offers two key functionalities (Fig. 1E and F). First, the inferred branch probabilities enable quantitative estimation of each cell’s differentiation potential, serving as a robust proxy for cellular plasticity. Second, by assigning each cell to the lineage with the highest posterior probability, BayesTraj facilitates the analysis of branch-specific gene expression dynamics along pseudotime, enabling the identification of genes with lineage-specific temporal regulation.

### 3.2 Evaluation on simulated datasets

**Figure 2.**
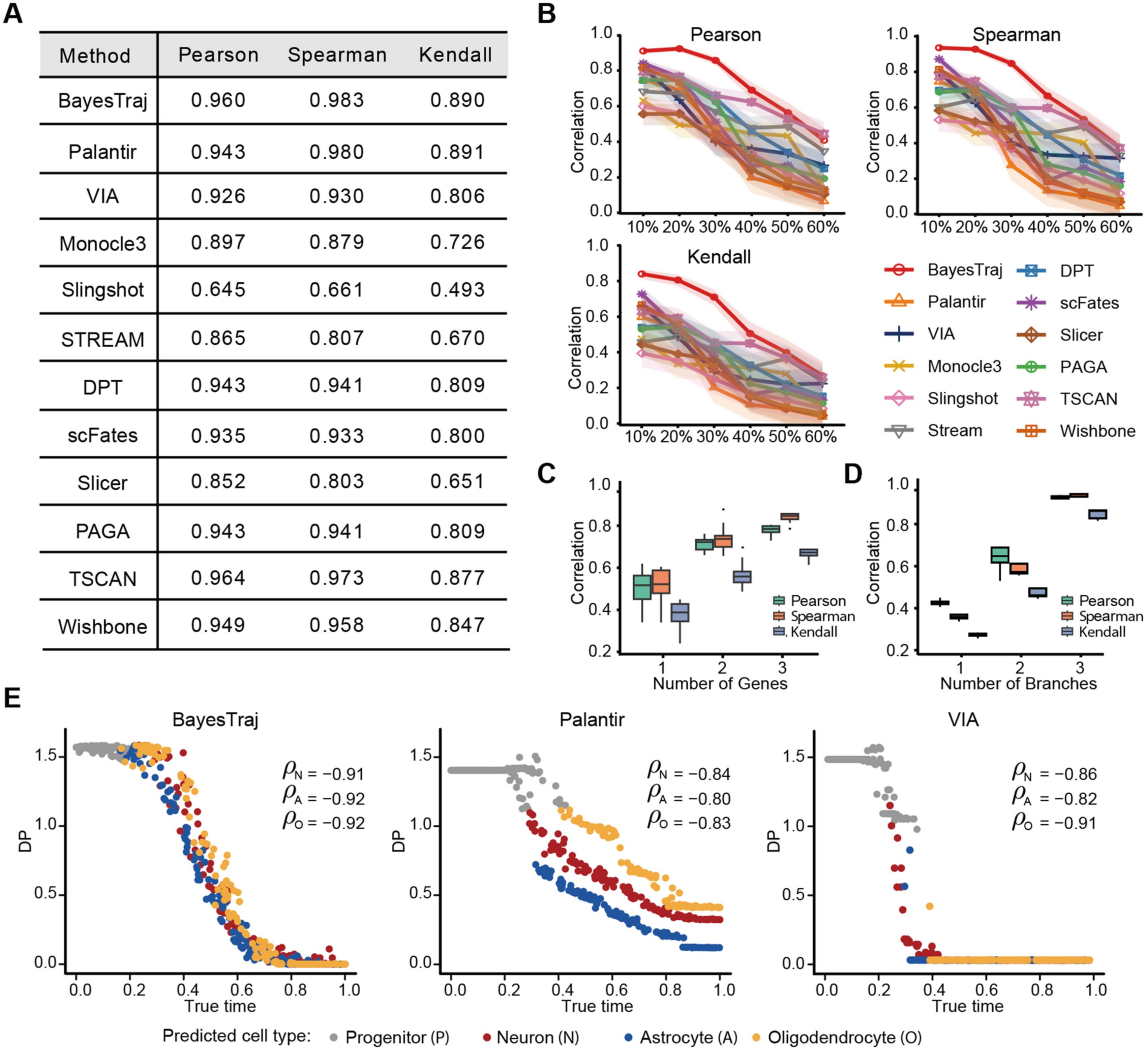
Comparison of BayesTraj with 11 other TI methods on simulated datasets: Palantir, VIA, Monocle3, Slingshot, STREAM, DPT, scFates, SLICER, PAGA, TSCAN, and Wishbone. (A) Global correlations (Pearson, Kendall, and Spearman) between inferred pseudotime and ground-truth time for each method. (B) Line plots with 95% confidence intervals illustrating how correlations change as the dropout rate increases from 10% to 60%. (C) Boxplots of correlations between inferred pseudotime and true time across 20 replicates as the marker‐gene panel size varies from one to three. (D) Boxplots of correlation metrics across 20 replicates when the three-branch prior is ablated to two or one branch. (E) Scatter plots of predicted differentiation potential (DP) versus true time for BayesTraj, Palantir, and VIA, with points colored by cell type according to a unified scheme (see Methods), and branch‐specific Spearman correlations are reported.

**Figure 3.**
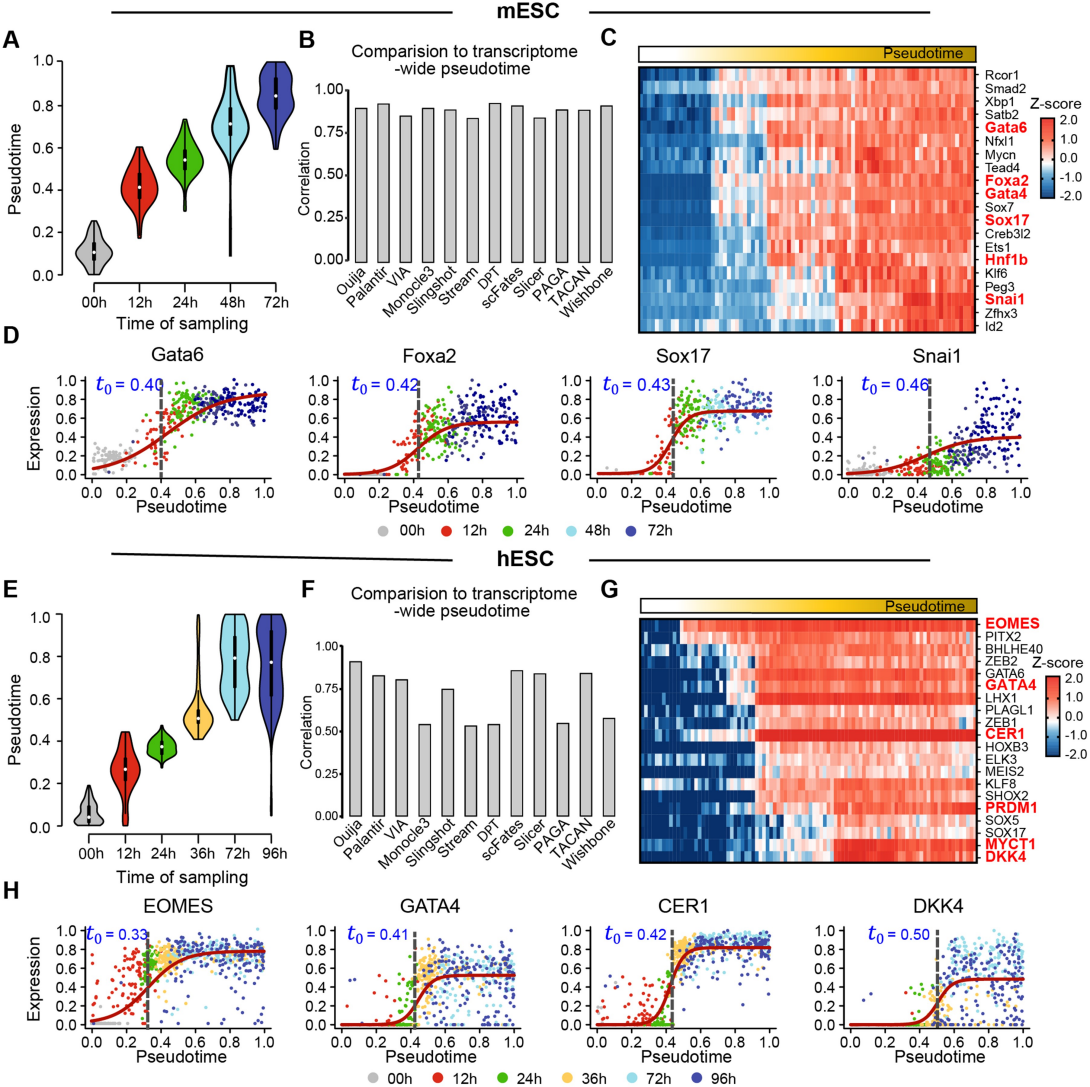
Performance evaluation of trajectory inference on directed differentiation datasets. (A) Violin plot showing the distribution of BayesTraj-inferred pseudotime versus true sampling times for single-cell progression from mESCs to definitive endoderm. (B) Comparison of pseudotime inferred by BayesTraj using only a small marker gene panel versus those obtained from Ouija and other transcriptome-wide TI methods (e.g. Palantir, VIA, Monocle3, Slingshot, STREAM, DPT, scFates, Slicer, PAGA, TSCAN, and Wishbone). (C) Heatmap of the top 20 branch-specific genes by Bayes Factor, showing their expression changes along the inferred pseudotime. (D) Expression of key mESC marker genes across inferred pseudotime. Solid red curves indicate BayesTraj’s fitted expression profiles and vertical dashed lines denote the inferred gene activation times. (E–H) Corresponding panels for the hESC dataset, analogous to (A–D).

We first evaluated BayesTraj on a series of simulated datasets (Fig. 1, available as supplementary data at *Bioinformatics* online) and benchmarked its performance against 11 state-of-the-art methods: Palantir, VIA, Monocle3, Slingshot, STREAM, DPT, scFates, SLICER, PAGA, TSCAN, and Wishbone. BayesTraj achieved the highest Spearman correlation with true time, as well as near-optimal Pearson and Kendall correlations (Fig. 2A, Fig. 2, available as supplementary data at *Bioinformatics* online). Even under 50% synthetic dropout, BayesTraj maintained high reconstruction accuracy, whereas competing algorithms exhibited substantial performance degradation (Fig. 2B). By varying the number of marker genes per branch from one to three, we found that even a single marker produced reasonable pseudotime estimates, with progressive improvements observed as additional markers were incorporated (Fig. 2C). In a lineage-prior ablation study, we progressively simplified the user-supplied branch topology from three lineages to one. BayesTraj still achieved reasonable accuracy, exhibited graceful performance degradation, and demonstrated robustness to incomplete branch information (Fig. 2D). The corresponding runtimes are reported in Fig. 3, available as supplementary data at *Bioinformatics* online.

**Figure 4.**
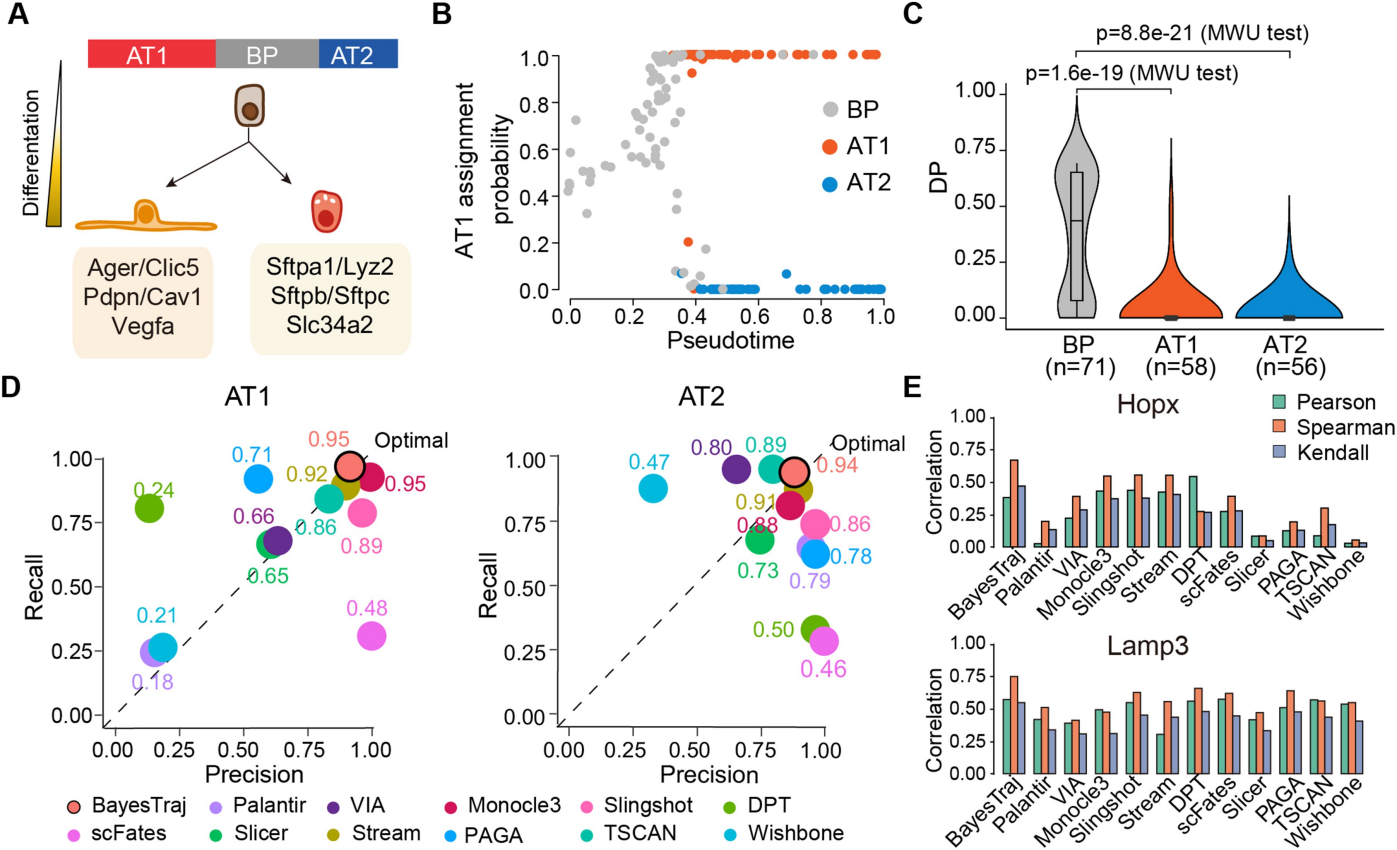
Performance evaluation on the lung dataset. (A) Schematic of the inferred bifurcation from the bipotent progenitor (BP) into two terminal fates: alveolar type I (AT1) and type II (AT2) cells, with representative marker genes shown for each lineage. (B) Scatter plot of branch‐assignment probabilities for AT1 fate versus BayesTraj-inferred pseudotime. Cells are colored by their annotated cell type. (C) Violin plot of differentiation potential (DP) estimated by BayesTraj for BP, AT1, and AT2 populations. Statistical significance between BP and each terminal state was evaluated by one-tailed Mann–Whitney U test. (D) Precision-recall plots comparing branch-assignment accuracy for AT1 and AT2 across 12 trajectory inference methods. Each point represents a method, with the embedded number indicating its ${\;F}_{1}$ score. (E) Bar plots of correlations between BayesTraj-inferred pseudotime and the expression of lineage-specific markers: Hopx for AT1 and Lamp3 for AT2.

Among the methods we compared, only BayesTraj, Palantir, and VIA provide per-cell probabilities of branch-assignment, which facilitate the quantification of differentiation potential. To ensure a fair comparison, we explicitly specified the same early cell as the starting point for Palantir and VIA. On probability simplex plots (Fig. 4, available as supplementary data at *Bioinformatics* online), BayesTraj traced a continuous trajectory from the center of the simplex toward the three terminal-fate vertices, closely recapitulating true developmental progression. In contrast, Palantir and VIA yielded nearly linear distributions, failing to capture gradual, non-linear transitions. When mapped onto the same simplex, BayesTraj’s differentiation potential estimates aligned well with the true progression. Quantitatively, BayesTraj achieved the strongest negative Spearman correlation between differentiation potential and true time across all three branches (Fig. 2E), underscoring its superior capacity to quantify cell-fate uncertainty.

### 3.3 BayesTraj captures hierarchies in directed differentiation

Although BayesTraj was primarily designed for multi-branch differentiation, it also demonstrated robust performance in directed differentiation systems. When applied to time-course datasets from mESC (Hayashi *et al.* 2018) and hESC (Chu *et al.* 2015), the inferred pseudotime showed high concordance with experimentally defined differentiation stages (Fig. 3A and E). In these single-lineage contexts, BayesTraj achieved a Spearman correlation of approximately 0.9 with Ouija, indicating that it effectively reduces to Ouija’s linear generative model when appropriate. Furthermore, pseudotime estimates showed strong agreement with transcriptome-wide methods, achieving Spearman correlations above 0.75 for mESC and above 0.5 for hESC in most comparisons (Fig. 3B and F).

**Figure 5.**
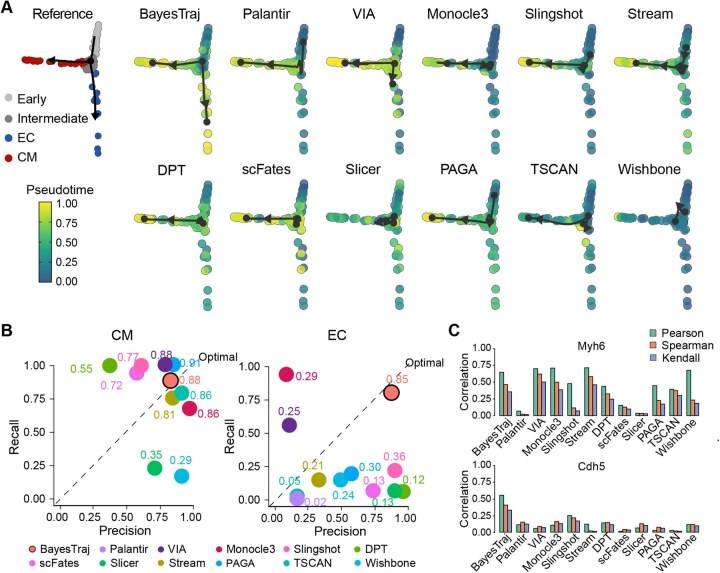
Performance evaluation on cardiac progenitor cell differentiation dataset. (A) Inferred trajectories from each algorithm projected onto a unified t-SNE embedding, with reference clusters: Early, Intermediate, endothelial (EC), and cardiomyocyte (CM). Subsequent panels superimpose inferred pseudotime as a continuous gradient, with arrows denoting the predicted direction of lineage progression. (B) Precision–recall scatter plots comparing the performance of TI methods. Each point represents one method and its ${\;F}_{1}$ score is labeled near each point. (C) Bar plots showing the correlation between inferred pseudotime and the expression of the marker genes Myh6 and Cdh5 along each of the two predicted trajectories.

To identify key regulators associated with directed differentiation, we ranked the top 20 genes by Bayes Factor (${BF}_{10}$), and ordered them by estimated activation times (Table 4, available as supplementary data at *Bioinformatics* online). Gene ontology analysis revealed significant enrichment for endoderm formation and cell-fate regulation (Fig. 5, available as supplementary data at *Bioinformatics* online). In the mESC dataset, Gata6, Foxa2, Gata4, and Sox17 were inferred to activate nearly simultaneously, followed by Snai1, consistent with early endodermal specification (Fig. 3C and D). In the hESC dataset, EOMES initiated a cascade of downstream activation involving GATA4, GATA6, and CER1, and later PRDM1, MYCT1, and DKK4, marking progression toward definitive endoderm (Fig. 3G and H). These results are consistent with previous developmental studies (Smith *et al.* 2010, Wamaitha *et al.* 2015, Prajapati *et al.* 2019, Scheibner *et al.* 2021, Schröder *et al.* 2025), thereby further substantiating the biological validity of our method.

### 3.4 BayesTraj reveals bifurcation dynamics in mouse lung differentiation

We applied BayesTraj to mouse lung scRNA-seq data (Treutlein *et al.* 2014) of bipotent progenitor (BP) differentiating into alveolar type I (AT1) and type II (AT2) cells (Fig. 4A). By leveraging canonical marker genes for AT1 and AT2 lineages, BayesTraj faithfully reconstructed the bifurcation event and resolved two distinct pseudotemporal trajectories (Fig. 4B). Differentiation potential, quantified as entropy of branch-assignment probabilities, decreased sharply from progenitors to both AT1 and AT2 lineages (Mann–Whitney U test $P<10^{-19}$; Fig. 4C).

**Figure 6.**
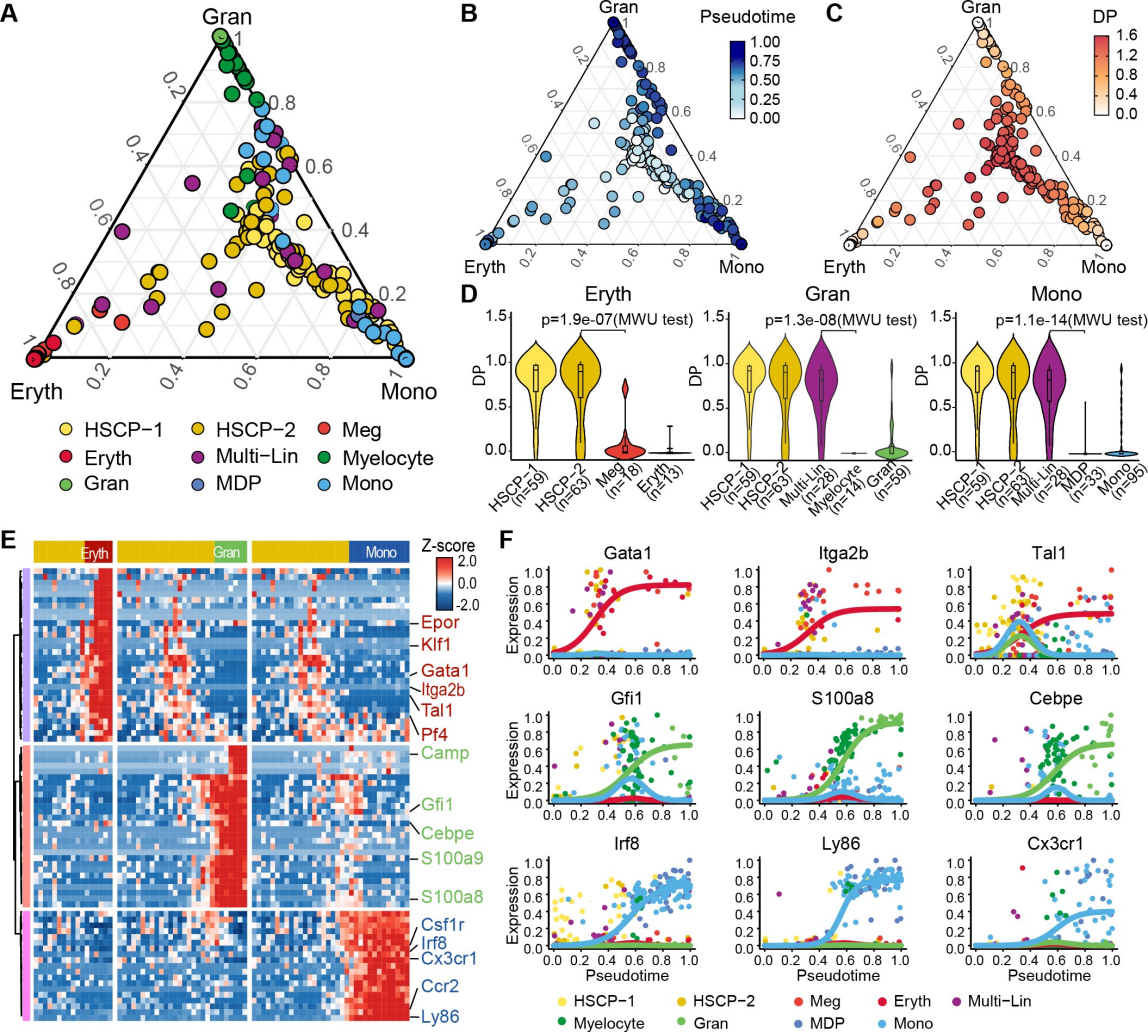
Performance evaluation on mouse hematopoietic stem cell differentiation dataset. (A) Probability simplex visualization of three developmental trajectories inferred by BayesTraj, colored by annotated cell-type labels from the original paper. (B and C) Simplex embedding colored by inferred pseudotime and differentiation potential (DP), illustrating progression from multipotent progenitors toward terminal fates. (D) Violin plots of DP distributions across cell types for the Eryth (left), Mono (right), and Gran (center) branches. Sample sizes for each cell‐type group are indicated below. Statistical significance for adjacent cell‐stage comparisons is denoted by one‐sided Mann–Whitney U test *P*‐value annotations. (E) Hierarchical clustering heatmaps showing the differential expression of branch-specific genes identified by BayesTraj. (F) Marker-gene expression dynamics along inferred pseudotime. Each scatter plot displays the expression of an individual marker gene across cells ordered by BayesTraj‐inferred pseudotime. Cells are colored by their annotated cell‐type labels. Solid lines represent the parametric fits estimated by BayesTraj, illustrating the dynamic activation and repression patterns of each marker along the differentiation trajectory.

Branch-dependent gene analysis revealed that AT1-specific genes were enriched for tube-development pathways, aligned with their role in alveolar maintenance, whereas AT2-specific genes were enriched for lipid metabolism processes, reflecting surfactant synthesis (Fig. 6 and Table 5, available as supplementary data at *Bioinformatics* online). A branch-restricted heatmap further confirmed that AT1- specific genes were exclusively upregulated along the AT1 lineage, and AT2-specific genes along the AT2 lineage.

Notably, we detected a subset of cells exhibiting a distinct intermediate state, characterized by the co-expression of lineage-specific markers from two divergent differentiation trajectories. These cells were found in the intermediate stages of BP cell differentiation into AT1 and AT2 cells, suggesting the presence of a transient bipotent state. This observation aligns with previous findings (Guo *et al.* 2019, Kobayashi *et al.* 2020) and highlights the existence of metastable, mixed-lineage cellular states that may play a critical role in orchestrating fate commitment (Sucre *et al.* 2023, Seasock *et al.* 2025).

To benchmark BayesTraj against other TI methods, we projected the trajectories inferred by each method onto a common Uniform Manifold Approximation and Projection (UMAP; McInnes *et al*. 2018) embedding generated via the dynwrap framework (Saelens *et al.* 2019). BayesTraj, along with Monocle3, Slingshot, STREAM, and TSCAN, accurately resolved the bifurcation of bipotent progenitors into AT1 and AT2 trajectories. In contrast, several other methods exhibited trajectory entanglement, misaligned pseudotime ordering, or inconsistent branch connectivity (Fig. 7, available as supplementary data at *Bioinformatics* online). Quantitatively, BayesTraj achieved the highest $F_{1}$ score for both AT1 and AT2 lineages, outperforming all other methods in branch-assignment accuracy. It also showed the strongest correlation between inferred pseudotime and the expression of Hopx (AT1 marker; Guo *et al.* 2018, Ota *et al.* 2018) and Lamp3 (AT2 marker; Salaun *et al.* 2004, Lunding *et al.* 2021), despite not using these genes during inference (Fig. 4D and E). These results highlight the strength of BayesTraj’s semi-supervised framework in integrating marker gene activation trends into pseudotime inference, leading to biologically faithful recapitulation of expected gene activation patterns. In terms of differentiation potential estimation, BayesTraj outperformed Palantir and VIA, revealing sharper separation between progenitor and committed states and greater sensitivity to fate transitions (Fig. 8, available as supplementary data at *Bioinformatics* online).

### 3.5 BayesTraj effectively reconstructs differentiation trajectories in imbalanced single-cell data

Our second bifurcation dataset was generated by profiling the differentiation process of murine Ils1+ cardiac progenitor cells (CPCs) into endothelial (EC) and cardiomyocyte (CM) lineages (Jia *et al.* 2018). A significant challenge in this data is the imbalance between the two terminal fates, with only 29 CM cells and 12 EC cells among 197 total cells. We found BayesTraj was the only method that accurately reconstructed both lineages with balanced precision and recall, while other methods either failed to uncover lineages or made incorrect predictions (Fig. 5A). For the CM lineage, most methods achieved a recall rate of 0.8 with minor precision differences, and our method ranked second in $F_{1}$ score, just behind Wishbone (Fig. 5B). However, most methods struggled with the EC lineage, whereas BayesTraj achieved balanced precision and recall, yielding an $F_{1}$ score of 0.85. Using Myh6 (Yan *et al.* 2015) and Cdh5 (Carmeliet *et al.* 1999) as independent validation genes, BayesTraj demonstrated moderate correlation in the CM lineage but achieved the highest correlation in the EC lineage (Fig. 5C). Other results for our method can be found in Figs 9–11 and Table 6, available as supplementary data at *Bioinformatics* online. These results demonstrate that BayesTraj effectively handles datasets with imbalanced cell distributions, providing accurate and robust trajectory inference in complex scenarios.

### 3.6 BayesTraj recapitulates mouse hematopoiesis differentiation

To further validate our method’s performance in a more complex differentiation system, we applied BayesTraj to analyze a published scRNA-seq dataset from Olsson et al. (2016) which focused on the mouse hematopoietic system. A total of 382 cells were isolated from the mouse wild-type hematopoietic system including stem/multipotent progenitor (LSK), common myeloid progenitor (CMP), granulocyte monocyte progenitor (GMP), and LKCD34+. BayesTraj successfully reconstructed the cell lineage hierarchy, as evidenced by the labels proposed in the original study (Fig. 6A). Hematopoietic stem cell progenitors (HSCPs) were localized near the center of the differentiation simplex, marking the origin of the differentiation. Terminally differentiated cells, such as erythroid (Eryth), granulocytic (Gran), and monocytic (Mono), are positioned near the vertices, reflecting fully committed fates. Intermediate populations, including Megakaryocytes (Meg), Multi-Lineage progenitors (Multi Lin), Myelocytes, and Monocyte-Dendritic progenitors (MDP) were distributed along trajectories connecting the center of the simplex to each terminal vertex, reflecting gradual shifts in lineage bias during differentiation. Pseudotime smoothly increased from multipotent HSPCs to lineage-committed cells, accompanied by a corresponding decline in differentiation potential (Fig. 6B and C). Kruskal–Wallis tests ($P<10^{-9}$) revealed significant differentiation in differentiation potential across annotated cell states in all three lineages. Pairwise Mann–Whitney U tests further confirmed substantial reductions at key lineage commitment stages (Fig. 6D). These results demonstrate that BayesTraj not only recapitulates the hierarchical organization of hematopoiesis but also pinpoints key stages in cell-fate determination, providing further insights into the dynamics of hematopoietic differentiation.

Applying a stringent evidence threshold (Bayes factor ${BF}_{10}>10$), BayesTraj identified 31, 33, and 19 branch-dependent genes for Eryth, Gran, and Mono, respectively (Table 7, available as supplementary data at *Bioinformatics* online). Clustering these branch-dependent genes by their expression profiles revealed distinct modules for each lineage (Fig. 6E and F). Gene ontology enrichment analysis confirmed that erythroid genes were enriched for erythropoiesis, granulocytic genes to immune-responses, and monocytic genes to macrophage activation and phagocytosis (Fig. 12, available as supplementary data at *Bioinformatics* online). These results demonstrate BayesTraj’s capacity to recover biologically coherent, lineage-specific regulatory programs.

In comparative benchmarking, only BayesTraj, Monocle3, and STREAM accurately recapitulated the reference topology, whereas the remaining methods predominantly captured only the Gran or Mono lineage (Fig. 13, available as supplementary data at *Bioinformatics* online). We evaluated branch-assignment accuracy using $F_{1}$ scores for each lineage. BayesTraj consistently achieved the highest precision and recall, approaching the “optimal” trade-off across three branches, outperforming the other methods. We also quantified concordance between inferred pseudotime and the expression of canonical marker genes not used in model inference (Mfsd2b for Eryth, Pilrb1 for Gran, Pyhin1 for Mono) (Banerjee *et al.* 2010, Chen *et al.* 2019, Ghaderi and Levkau 2023). BayesTraj exhibited strong correlations in all three cases, highlighting the reliability of its predicted pseudotime and its robust recovery of lineage-specific expression dynamics. Regarding differentiation potential estimation, we observed that, although Palantir exhibited significant declines in differentiation potential along the Gran or Mono branches, their Kruskal–Wallis tests were not significant for the Eryth branch (Fig. 14, available as supplementary data at *Bioinformatics* online). This suggests that their entropy-based potential scores fail to discriminate between multipotent early and late erythroid states, whereas BayesTraj more effectively captures the continuous decline in potency across all lineages.

## 4 Discussion

In this study, we introduce BayesTraj, a Bayesian generative model that incorporates a predefined lineage topology and marker-gene dynamics as priors to reconstruct single-cell developmental trajectories from scRNA-seq data. By fitting parametric curves to a small panel of marker genes within each user-specified branch, BayesTraj produces smooth pseudotime orderings and coherent differentiation-potential profiles. Its semi-supervised framework constrains inference to biologically plausible lineages, thereby attenuates technical noise and data sparsity, and enforces monotonic marker-gene expression changes along each trajectory. Moreover, the fully probabilistic model jointly infers pseudotime, branch-assignment probabilities, and gene dynamic parameters, yielding “soft” assignments that reveal intermediate or metastable states and quantify cellular plasticity via Shannon entropy. In contrast, purely unsupervised methods that rely only on cell–cell similarity often fail to resolve such intermediate states and are more sensitive to noise.

Despite these strengths, BayesTraj has several notable limitations. First, accuracy depends on user-provided priors. If the supplied lineage topology is incorrect or incomplete, the model may be biased toward suboptimal trajectories. Future work could explore empirical Bayes strategies to dynamically recalibrate lineage priors based on the observed data, thereby reducing reliance on perfectly specified topology. Second, the current framework restricts marker-gene dynamics to simple parametric templates (e.g. sigmoidal or single-pulse function), which may not capture more complex transcriptional dynamics such as multimodal or oscillatory expression. Integrating nonparametric methods or neural ODE models could broaden BayesTraj’s capacity to model diverse kinetic patterns. Finally, BayesTraj uses HMC sampling to approximate the joint posterior, offering asymptotically exact estimates but at the cost of substantial computational resources and slower convergence on large, heterogeneous datasets. Future implementations could adopt variational Bayesian inference or GPU-accelerated sampling to improve scalability and convergence speed.

In summary, BayesTraj provides a principled and interpretable framework for trajectory inference by integrating marker-gene biological priors into a hierarchical Bayesian model. By explicitly modeling pseudotime, lineage assignments, and gene activation dynamics, BayesTraj enables accurate reconstruction of complex differentiation hierarchies, robust quantification of cell-fate potential, and systematic identification of branch-specific regulatory programs. These capabilities advance the computational dissection of cellular differentiation and lay the groundwork for future extensions that incorporate multi-modal single-cell data, such as spatial transcriptomics and epigenomic measurements.

## Supplementary Material

btaf454_Supplementary_Data

## Data Availability

The data underlying this article are available in the article and in its online supplementary material.
