# Supplementary material for: A semi-supervised Bayesian approach for marker gene trajectory inference from single-cell RNA-seq data
Source: Bioinformatics. 2025 Aug 13;41(9):btaf454. doi: 10.1093/bioinformatics/btaf454 (PMC12410927; doi:10.1093/bioinformatics/btaf454)
Supplement: btaf454_Supplementary_Data [file btaf454_supplementary_data.docx]

**Supporting Information**

A Semi-Supervised Bayesian Approach for Marker Gene Trajectory Inference from Single-Cell RNA-Seq Data

Junchao Wang^1,†^, Ling Sun^1,†^, Nana Wei^2,†^, Yisheng Huang^1^, Naiqian Zhang^1, *^

^1^School of Mathematics and Statistics, Shandong University, Weihai, 264209 China and ^2^Key laboratory of Carcinogenesis and Translational Research (Ministry of Education/Beijing), Department of Lymphoma, Peking University Cancer Hospital & Institute, Beijing, 100142, China

^*^To whom correspondence should be addressed.

^†^The authors wish it to be known that, in their opinion, the first three authors should be regarded as Joint First Authors.

Contents

[S1. Supplementary figures 1](#_Toc203838194)

[S2. Supplementary tables 15](#_Toc203838195)

[S3. Simulating the neuronal differentiation trajectory 23](#_Toc203838196)

[S4. Data Preprocessing 25](#_Toc203838197)

[S5. Algorithmic Summaries of Trajectory Inference Methods 26](#_Toc203838198)

[SI References 29](#_Toc203838199)

S1. Supplementary figures


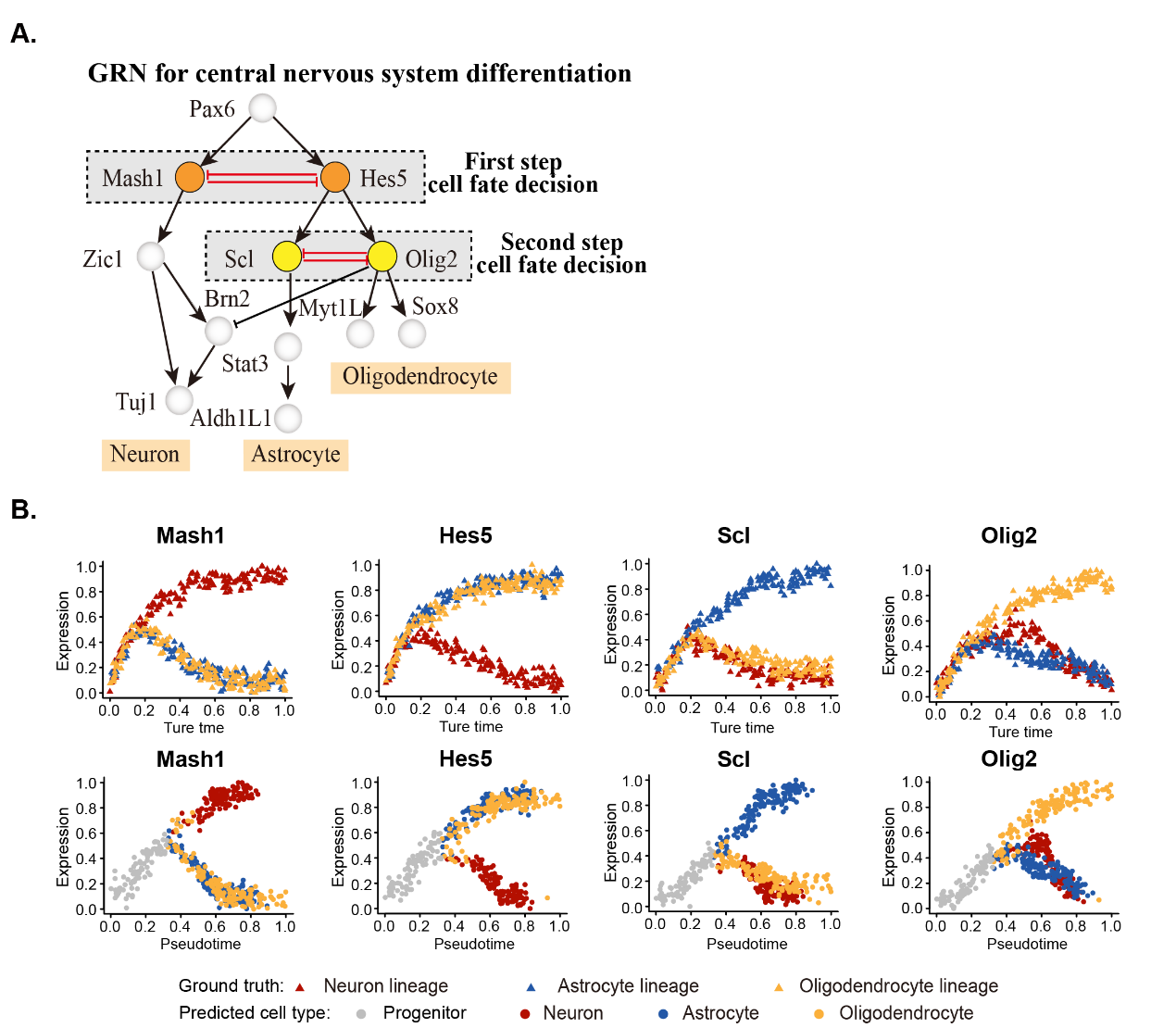


Fig. S1. Simulated Differentiation Trajectories and Gene Expression Patterns in the Central Nervous System. (A) Schematic of the central nervous system gene regulatory network, featuring two mutually inhibitory transcription factor pairs (Mash1/Hes5 and Scl/Olig2) that drive three-way cell fate specification. (B) Expression dynamics of four lineage-specific transcriptional factors plotted against true time (top) and BayesTraj inferred pseuodotime (bottom). In the upper panels, cells are colored by their annotated lineage, whereas in the lower panels, cells are colored to reflect BayesTraj’s predicted cell states.


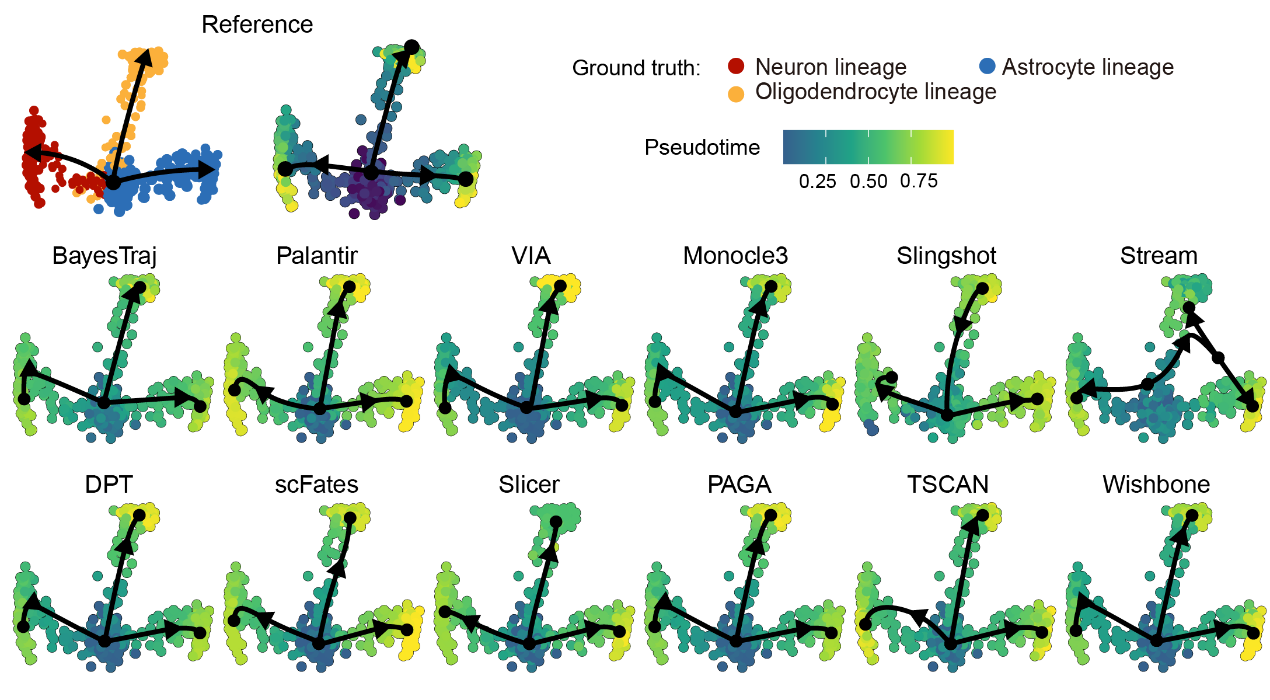


Fig. S2. Trajectories of the simulated dataset inferred by BayesTraj and other methods. Inferred trajectories from each algorithm projected onto the common diffusion‐map embedding. The reference panel displays ground-truth cell states and cell different times. In the subsequent panels, cells are colored by inferred pseudotime and arrows indicate the predicted direction of lineage progression.


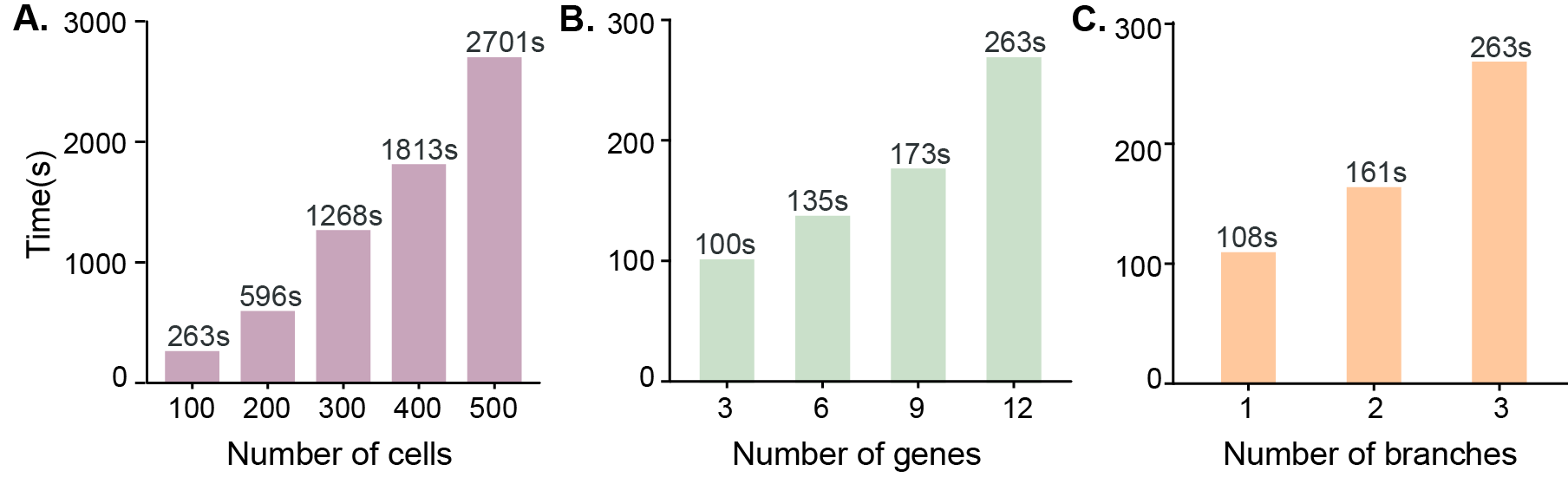


Fig. S3 Runtime performance of BayesTraj. (A) Runtime scales linearly with cell number (12 genes, 3 branches fixed). (B) Runtime scales linearly with gene number (100 cells, 3 branches fixed). (C) Runtime increases moderately with branch number (100 cells, 12 genes fixed).


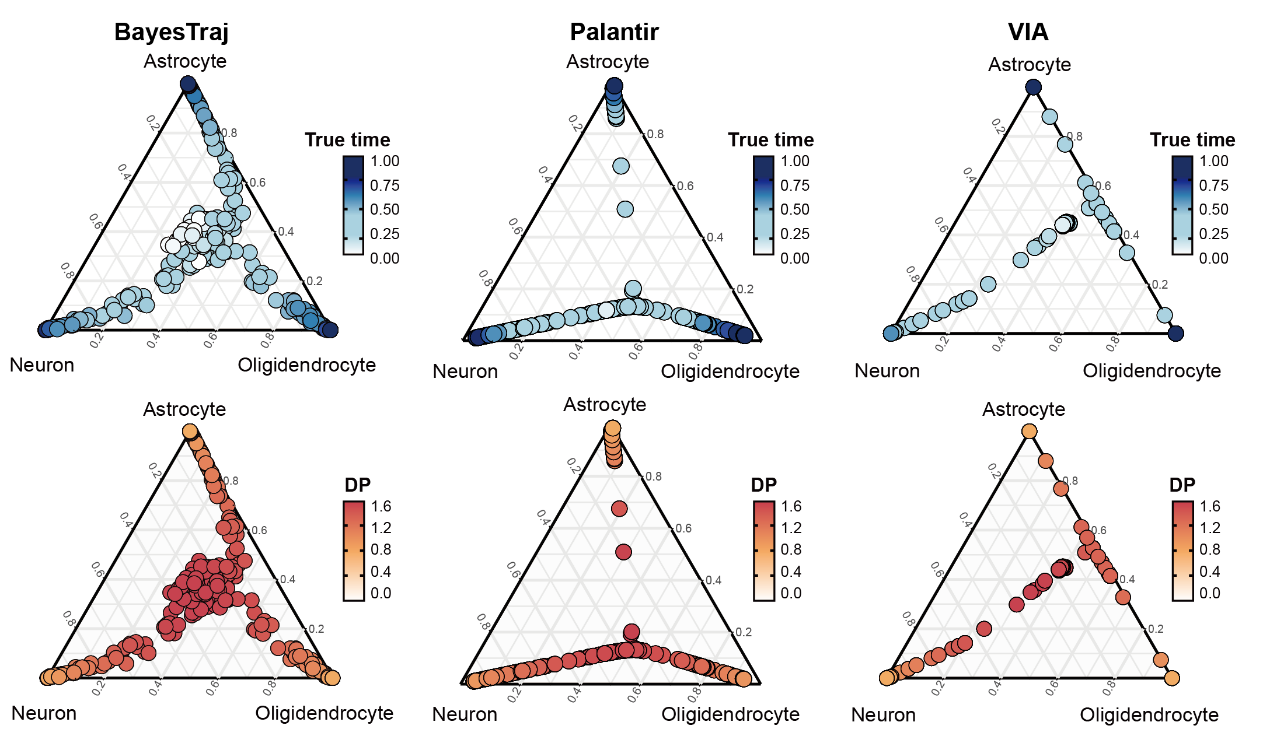


Fig. S4. Probability‐simplex visualization of branch‐assignment outputs for BayesTraj, Palantir and VIA. In the top panels, cells are plotted on the fate simplex according to their probabilities of belonging to the Neuron, Astrocyte, and Oligodendrocyte branches, with point color indicating true developmental time; in the bottom panels, the same coordinates are colored by differentiation potential (DP).


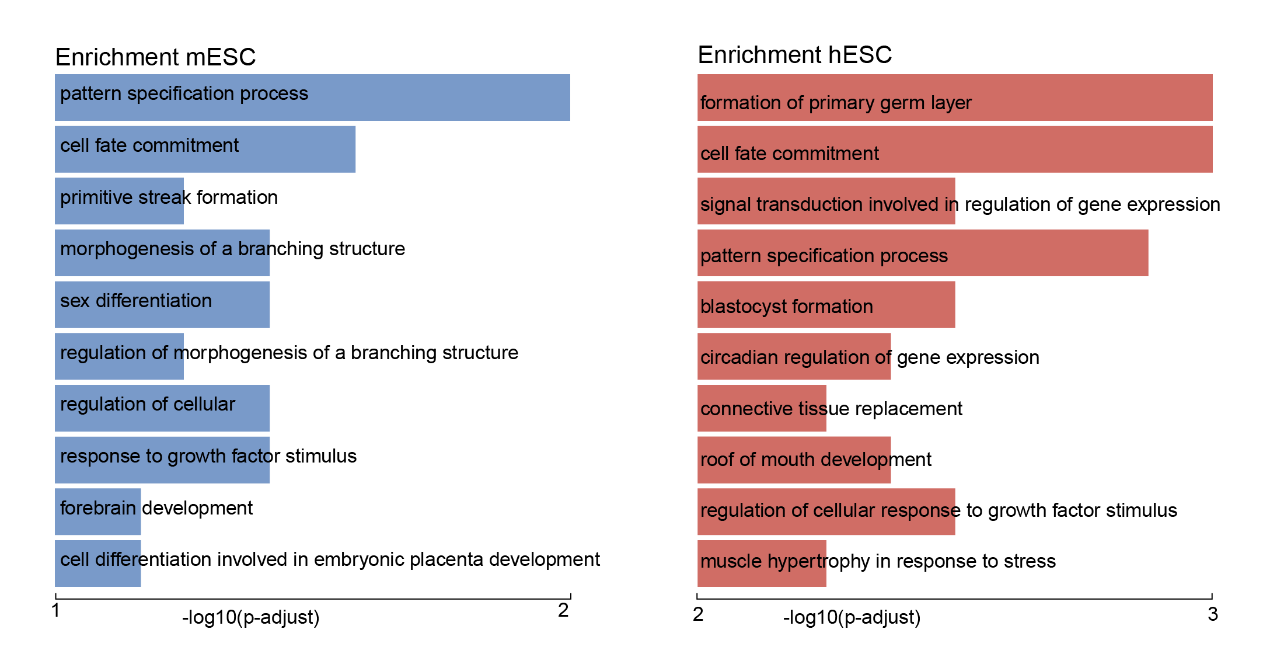


Fig. S5. Gene Ontology enrichment of the top 20 branch‐specific genes identified by BayesTraj in mESC and hESC.


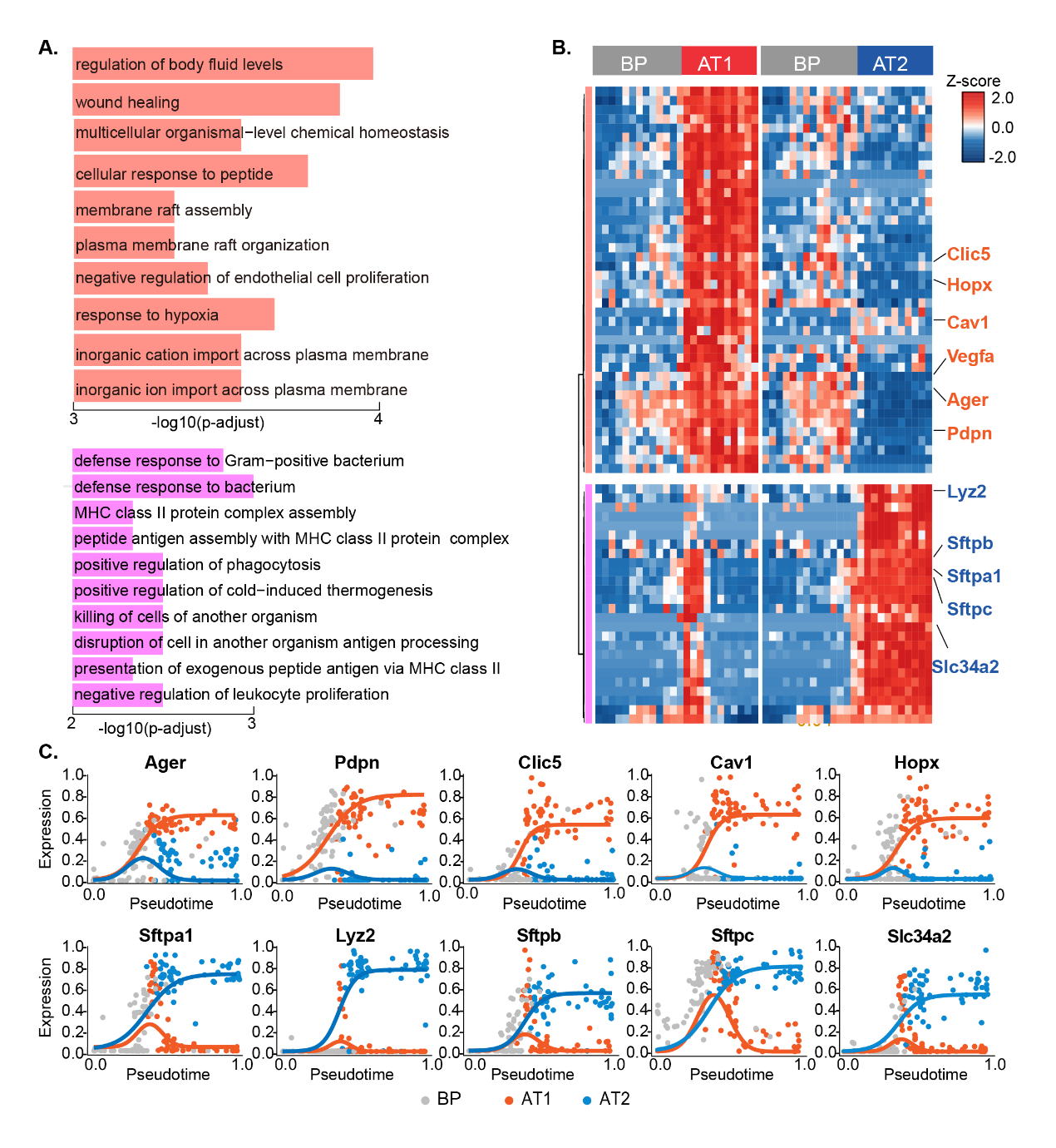


Fig. S6. Enrichment Analysis and Temporal Dynamics of Branch-Specific Marker Genes Inferred by BayesTraj. (A) Gene‐Ontology enrichment of the top branch‐specific markers, shown as log₁₀ (adjusted p‐value) barplots. (B) Hierarchical clustering heatmap of Z‐scored expression for the top branch‐specific genes, with cells grouped by annotated cell types (BP, AT1, AT2). (C) Marker gene expression dynamics along inferred pseudotime. Each scatter plot displays the expression levels of an individual marker gene across cells ordered by BayesTraj‐inferred pseudotime. Cells are colored by their annotated cell‐type labels (BP, AT1, AT2). Solid lines represent the parametric fits estimated by BayesTraj, illustrating the dynamic activation and repression patterns of each marker along the differentiation trajectory.


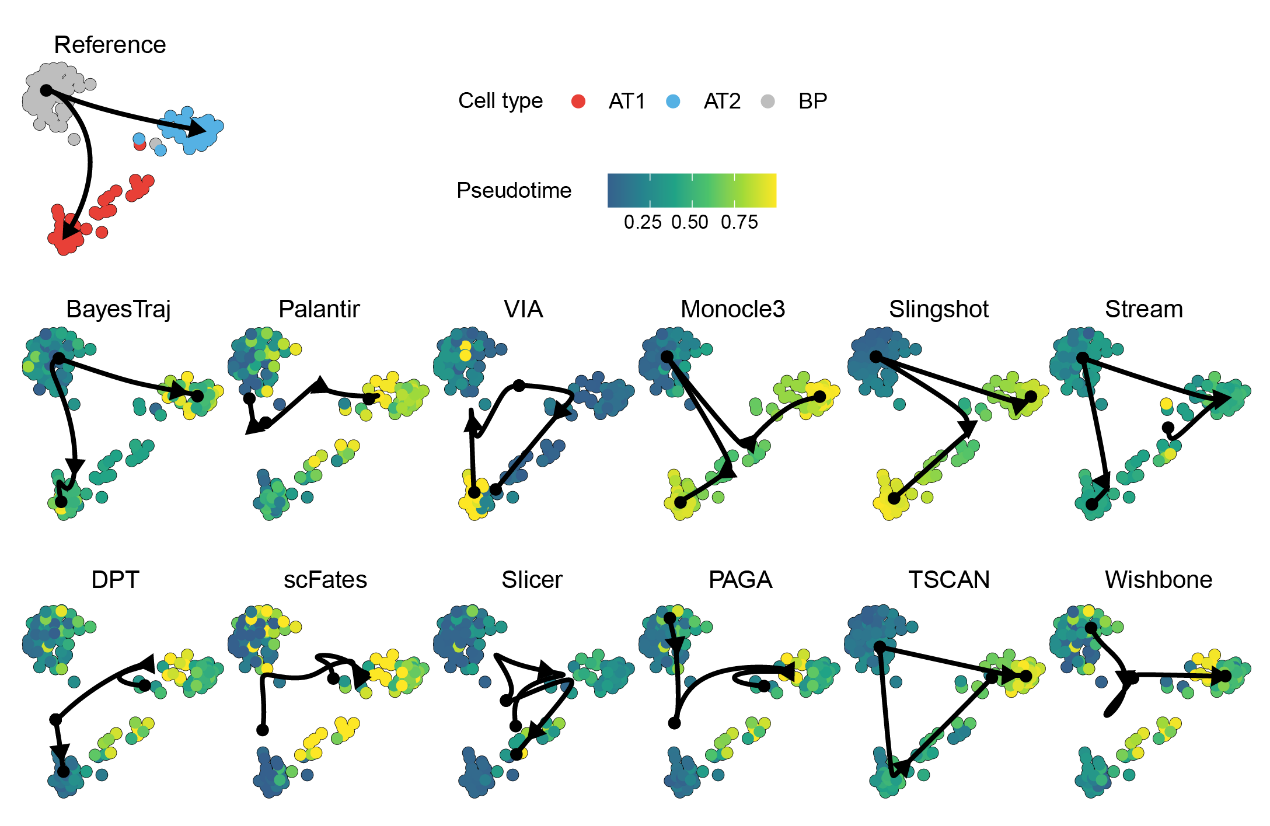


Fig. S7. Trajectories of the lung dataset inferred by BayesTraj and other methods.


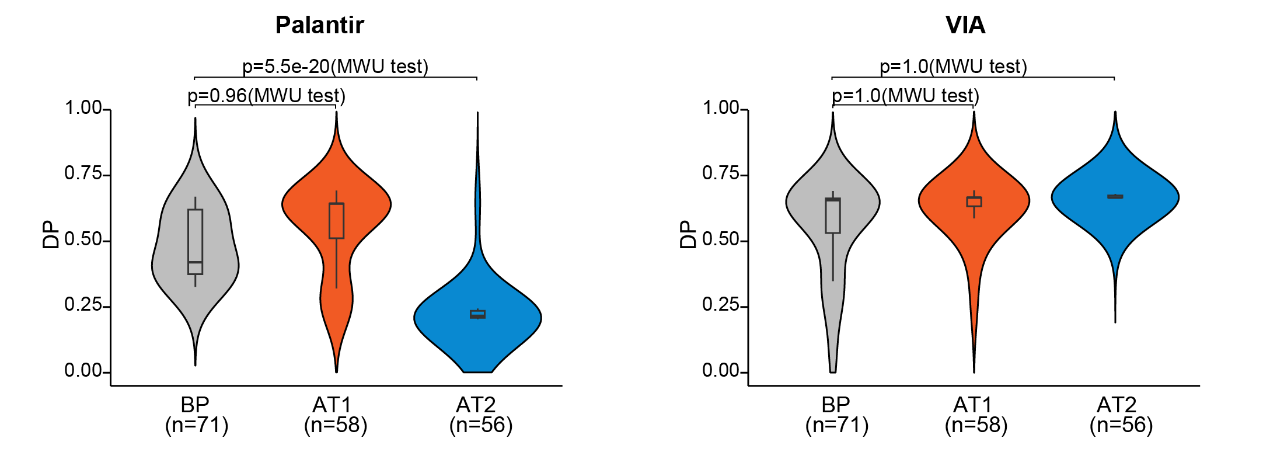


**Fig. S8. Differentiation-potential (DP) distributions inferred by Palantir and VIA.** Statistical significance between BP and each alternative fate was evaluated by one-tailed Mann-Whitney U test.


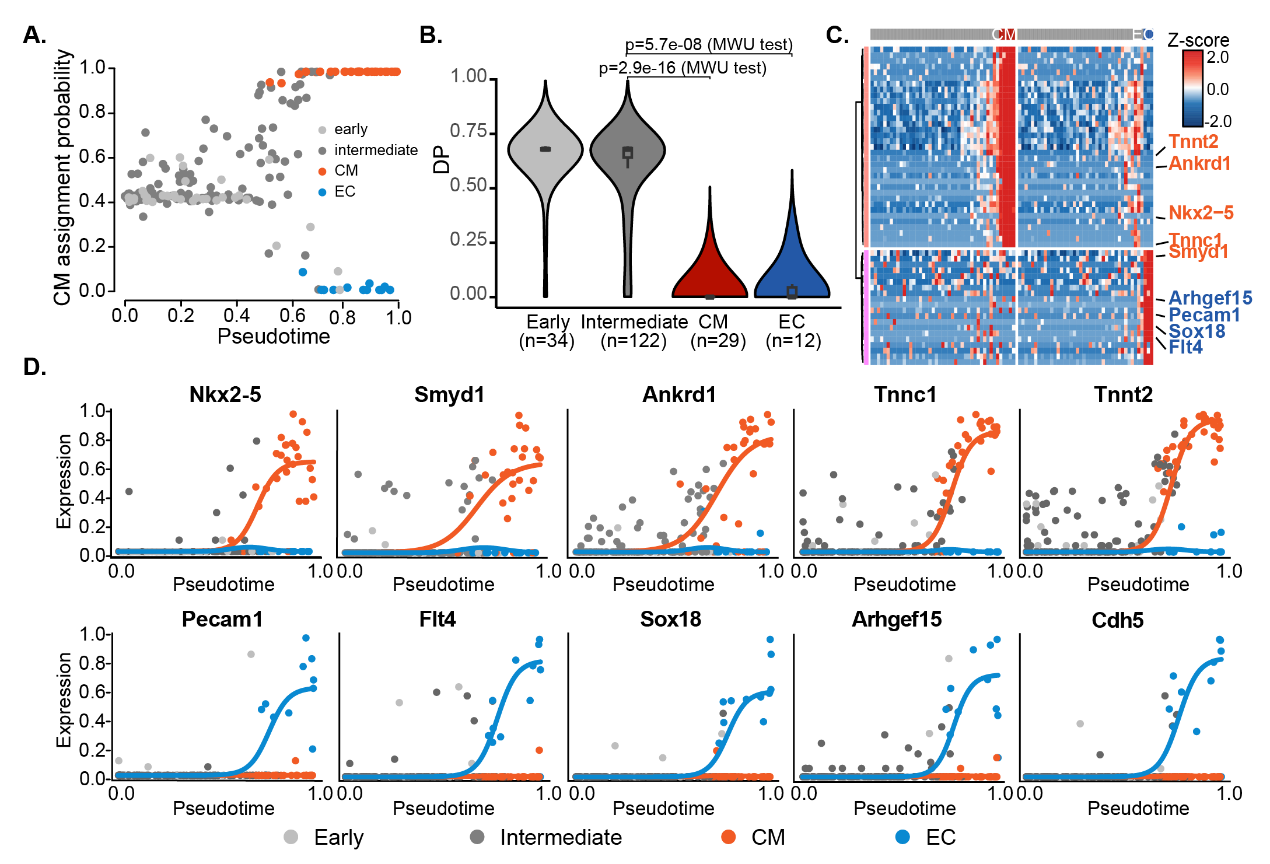


Fig. S9. BayesTraj application to cardiac progenitor cell differentiation dataset. (A) CM branch assignment probability versus inferred pseudotime using BayesTraj. Cells are coloured by their annotated cell‐type labels (early, intermediate, CM, EC). (B) Violin plots of differentiation potential (DP) estimated by BayesTraj for progenitor cells and two alternative trajectories (CM, EC). Adjacent stage comparisons were evaluated by one‐tailed Mann–Whitney U tests, with significant differences annotated. (C) Hierarchical clustering heatmap showing the differential expression of branch-specific genes identified by BayesTraj. (D) Marker gene expression dynamics along inferred pseudotime. Each scatter plot displays the expression levels of an individual marker gene across cells ordered by BayesTraj‐inferred pseudotime. Solid lines represent BayesTraj fits based on the posterior mode of the estimated gene behavior parameters in the both lineages.


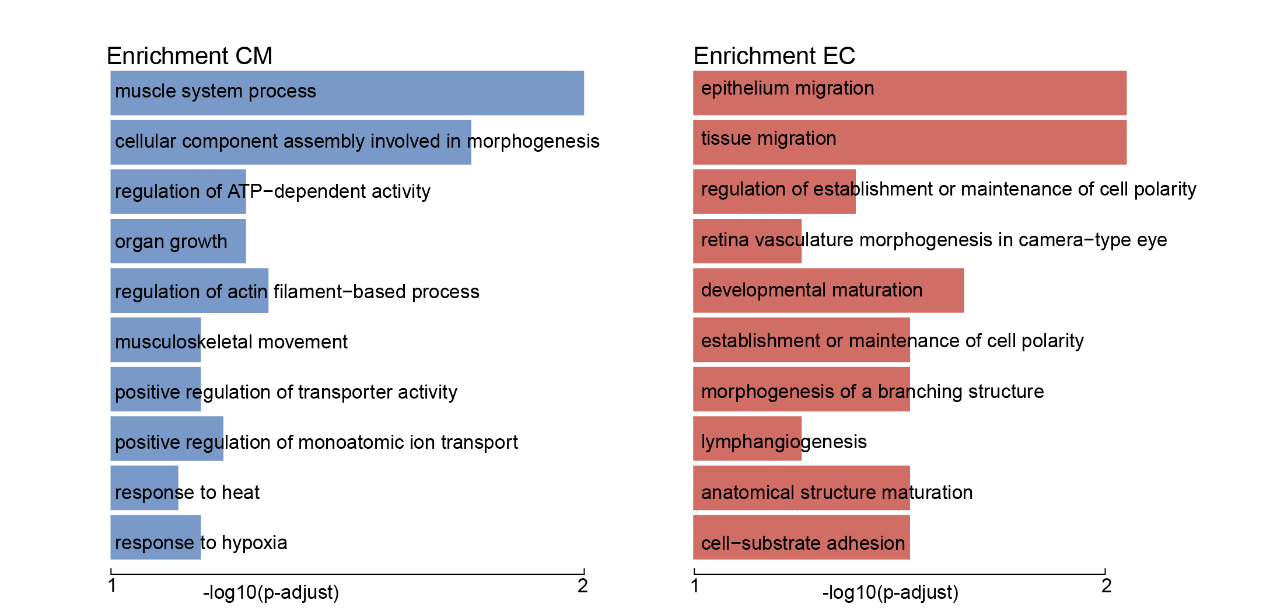


Fig. S10. Gene function enrichment of branch‐specific genes identified by BayesTraj in CM and EC.


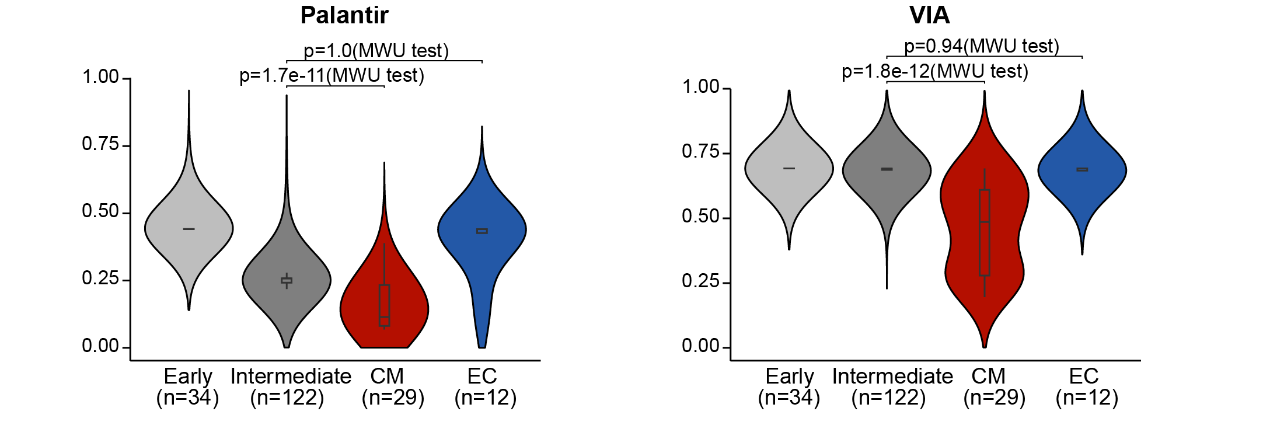


**Fig. S11. Violin plots of differentiation potential (DP) estimated by Palantir and VIA.** Adjacent stage comparisons were evaluated by one‐tailed Mann–Whitney U tests, with significant differences annotated.


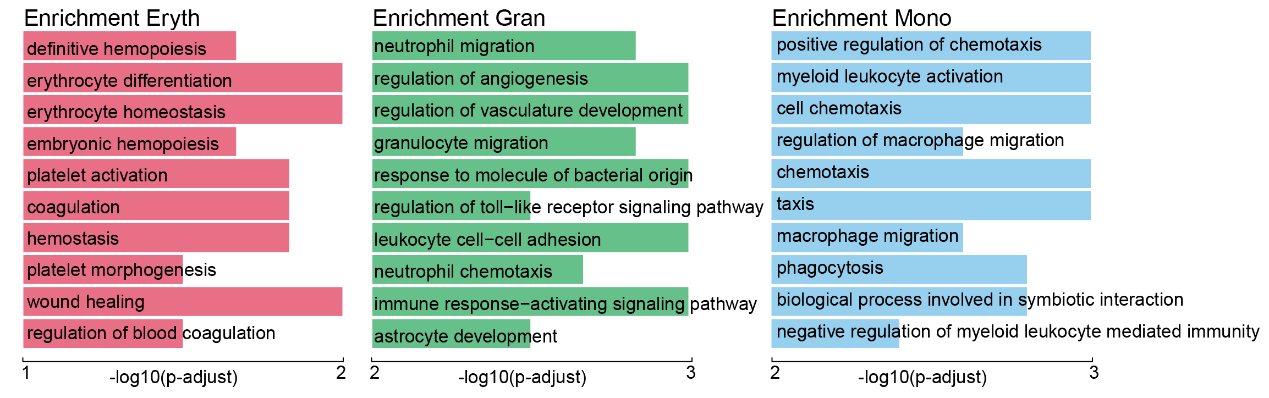


Fig. S12. Gene function enrichment of branch‐specific genes identified by BayesTraj in Eryth, Gran and Mono.


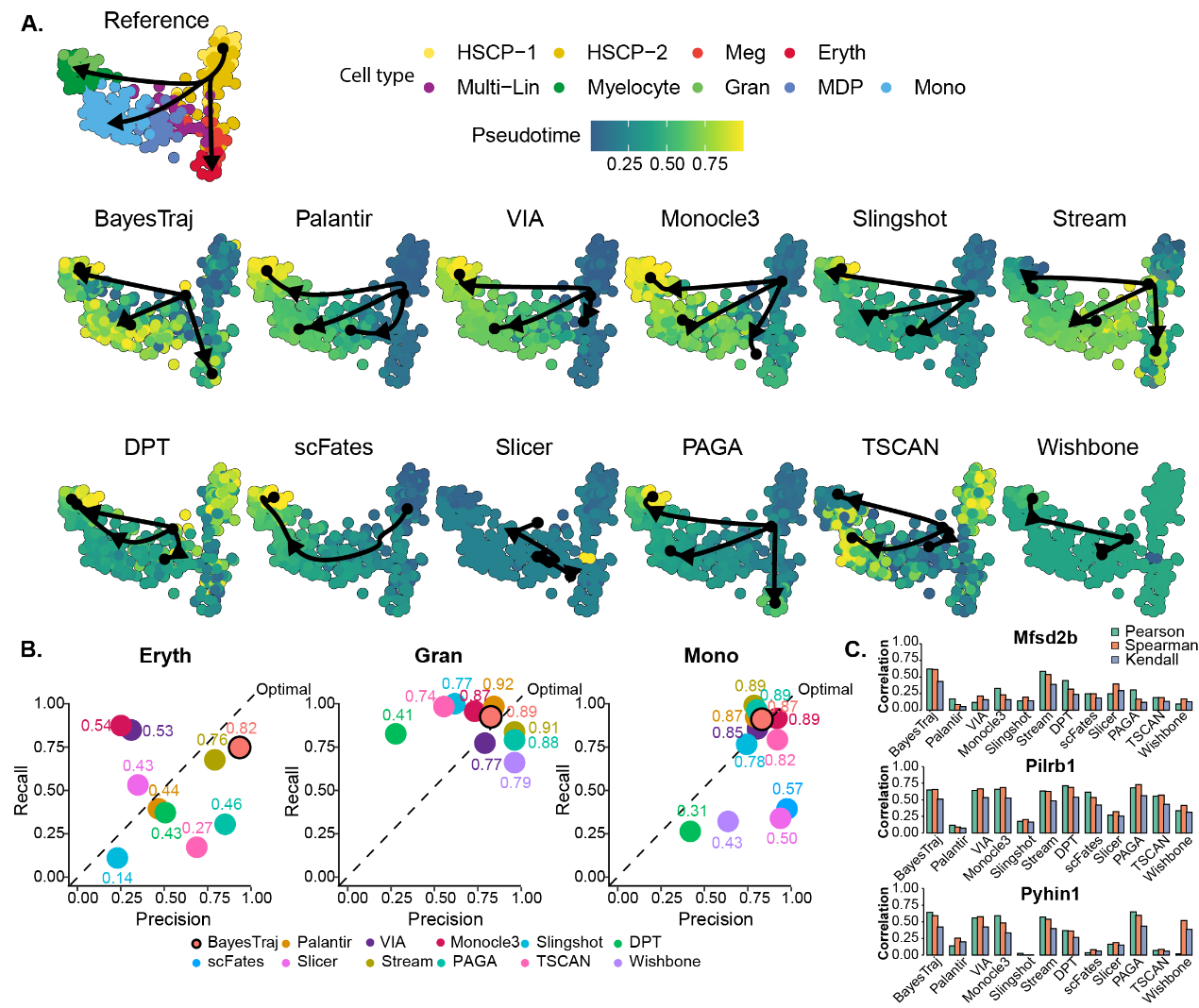


Fig. S13. BayesTraj application to mouse hematopoietic stem cell differentiation dataset. (A) Inferred trajectories from each algorithm projected onto the common diffusion‐map embedding. (B) Precision–recall scatter plots comparing the performance of each trajectory‐inference algorithm across individual lineage branches. Each circle represents one method and its F1 score is labeled near each circle. (C) Bar plots of the correlation between inferred pseudotime and the expression levels of marker genes Mfsd2b, Pilrb1, and Pyhin1 along each of the two predicted trajectories.


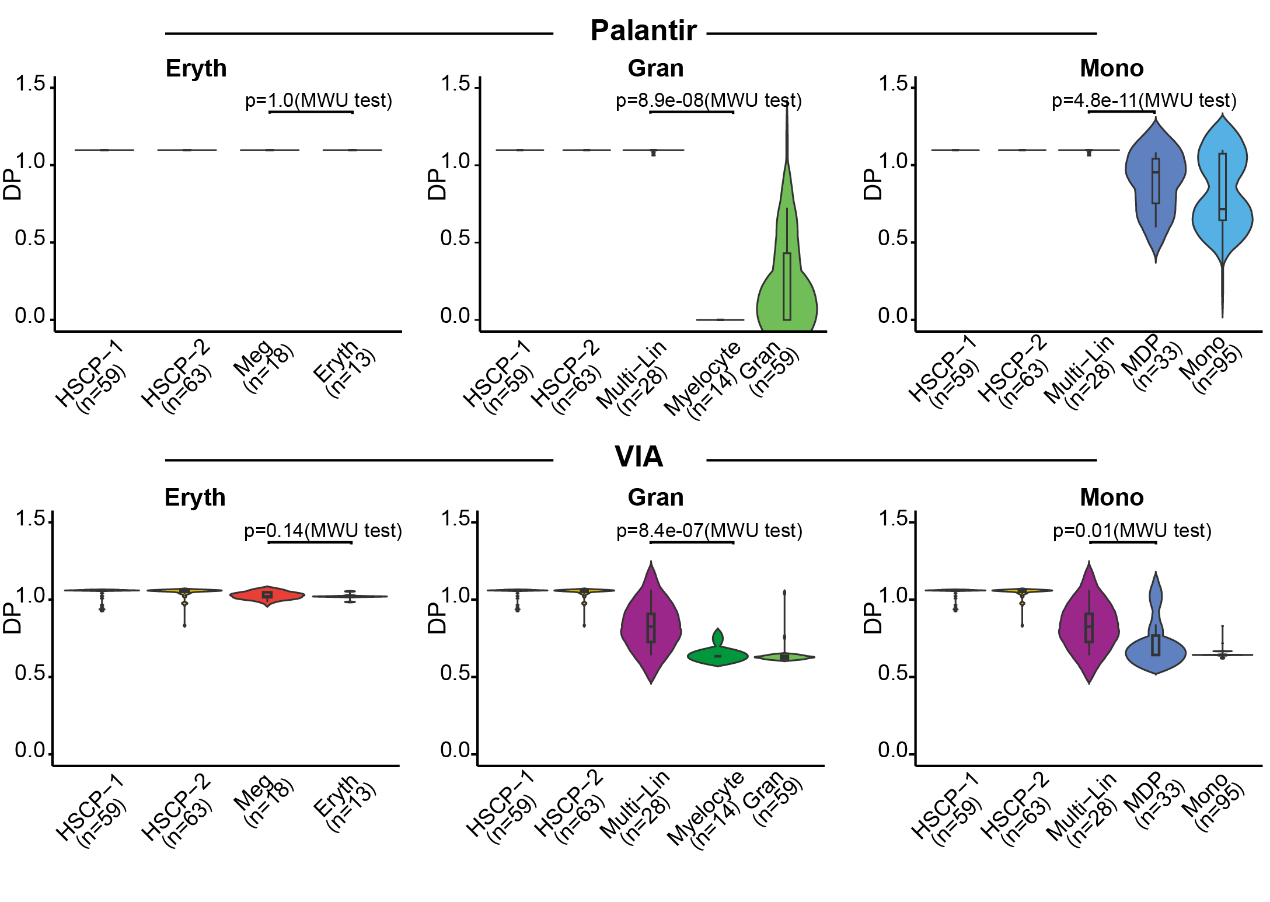


**Fig. S14. Violin plots show DP for successive cell states, estimated with Palantir and VIA.** Brackets give one-tailed Mann-Whitney U P-values comparing adjacent stages.

# S2. Supplementary tables

| **datasets** | **Single cell modality** | **Organism** | **Number of cells** | **Time series** | **Biological Process** | **Reference** |
| --- | --- | --- | --- | --- | --- | --- |
| 1.Mouse Embryonic Stem Cell (mESC) | scRNA-seq | Mouse | 421 cells | 0h,12h,24h,  48h,72h | mESC differentiation to DE | (Hayashi, et al., 2018) |
| 2.Human Embryonic Stem Cell (hESC) | scRNA-seq | Human | 758 cells | 0h,12h,24h,  36h,72h,96h | hESC  differentiation to DE | (Chu, et al., 2015) |
| 3.Lung Epithelium | scRNA-seq | Mouse | 185 cells | E14.5Adult | Lung development | (Treutlein, et al., 2014) |
| 4.Cardiac Progenitor | scRNA-seq | Mouse | 197 cells | E7.5,E9.5 | Cardiac development | (Jia, et al., 2018) |
| 5.Hematopoetsis | scRNA-seq | Mouse | 382 cells | E16.5 | Cardiac lineage specification | (Olsson, et al., 2016) |

|  |
| --- |

Table S1. Summary of datasets employed in the present study.

Table S2. Markers used in the five datasets studies.

| **Dataset** | **Category** | **Markers** |
| --- | --- | --- |
| **mESC** | Switch-like | Gata4, Sox17, Foxa2, Gata6, Id2, Snai1 |
|  | Transient | Foxh1, Foxa1, Gata1, Six1, Hmga2, Runx1 |
| **hESC** | Switch-like | EOMES, CER1, GATA4, DKK4, MYCT1, PRDM1 |
|  | Transient | CDX1, MSX2, T |
| **Lung Epithelium** | AT1 | Clic5, Pdpn, Cav1, Ager, Hopx, Vegfa |
|  | AT2 | Sftpa1, Lyz2, Sftpb, Sftpc, Slc34a2 |
| **Cardiac Progenitor** | EC | Sox18, Flt4, Arhgef15, Pecam1 |
|  | CM | Tnnt2, Nkx2-5, Ankrd1, Smyd1, Tnnc1 |
| **Hematopoiesis** | Eryth | Gata1, Klf1, Epor, Itga2b, Pf4, Tal1 |
|  | Gran | S100a8, S100a9, Gfi1, Camp, Cebpe |
|  | Mono | Ly86, Irf8, Ccr2, Cx3cr1 |

**Table S3. General comparison of the state-of-the-art TI methods.**

| **Method** | **Scalability** | **Complex Topology** | **Biological Prior Integration** | **Branch Probability Assignment** | **Reference** |  |  |  |
| --- | --- | --- | --- | --- | --- | --- | --- | --- |
| BayesTraj | Low | No | Marker genes  No. of branches | Yes |  |  |  |  |
| Ouija | Low | No | Marker genes | No | (Campbell and Yau, 2019) |  |  |  |
| Palantir | High | No | Early cell(s)  Terminal states | Yes | (Setty, et al., 2019) |  |  |  |
| VIA | High | Yes | Root cell | No | (Stassen, et al., 2021) |  |  |  |
| Monocle3 | High | No | Root cell | No | (Cao, et al., 2019) |  |  |  |
| Slingshot | Medium | No | No | No | (Street, et al., 2018) |  |  |  |
| STREAM | | High | No | No | No | (Chen, et al., 2019) |  |  |
| DPT | | | High | No | No | No | (Haghverdi, et al., 2016) |  |
| scFates | Medium | Yes | Root cell | No | (Faure, et al., 2022) |  |  |  |
| Slicer | | | | High | Yes | Start cell(s) | No | (Welch, et al., 2016) |
| PAGA | High | Yes | Start cell(s) | No | (Wolf, et al., 2019) |  |  |  |
| TSCAN | High | No | No | No | (Ji and Ji, 2016) |  |  |  |
| Wishbone | High | No | Start cell(s) | No | (Setty, et al., 2016) |  |  |  |

Many cutting-edge trajectory inference (TI) methods are capable of automating lineage-related analyses. However, based on extensive performance evaluations, these methods often encounter significant challenges when applied to increasingly large, diverse (multi-omic), and complex single-cell datasets. The primary challenges include:

**Scalability criteria for the haematopoiesis dataset (382 cells × 23,955 genes)**

- **High:** runtime < 1 minute
- **Medium:** runtime 1-10 minutes
- **Low:** runtime > 10 minutes

**Complex topology**: Capturing diverse trajectory structures, including cyclic, disconnected, hybrid, and tree-like topologies.

**Biological Prior Integration:** Uses biological knowledge, such as marker genes and lineage relationships, to guide trajectory inference, improving accuracy and interpretability, especially in complex or imbalanced systems.

**Branch Probability Assignment:** Assigns probabilistic weights to different branching paths in the trajectory, quantifying the likelihood of cells following specific developmental routes.

**Table S4. Branch-Specific Top 20 Genes Sorted by Bayesian Factors.**

| mESC | hESC |
| --- | --- |
| Gata4  Sox17  Gata6  Creb3l2  Foxa2  Creb3  Hnf1b  Smad2  Zfhx3  Carhsp1  Xbp1  Sox7  Nfxl1  Snai1  Rcor1  Hopx  Ets1  Peg3  Klf6  Cers2 | LHX1  GATA6  EOMES  SHOX2  PRDM1  GATA4  KLF8  BHLHE40  IRX3  PGR  HOXB3  SOX5  OTX2  ZEB2  ZEB1  ZNF521  JUND  SOX17  RAX2  MEIS2 |

**Table S5. Branch-Specific Top 20 Genes Sorted by Bayesian Factors.**

| Lung | |
| --- | --- |
| AT1 | AT2 |
| Clic5  Ager  Lgals3  Vegfa  Cav1  Gprc5a  Hopx  Clic3  Sec14l3  Ahnak  Lmo7  Pdpn  Tspan8  Akap5  Msln  Rtkn2  Igfbp6  Tmem213  Tmem37  Emp2 | Slc34a2  Lyz2  Sftpa1  Sftpd  Scd1  Chi3l1  Cxcl15  Lamp3  Sftpb  Hc  Cd36  S100g  Sftpc  Cbr2  H2-Aa  Rnase4  Lcn2  H2-Ab1  Il33  Atp6v1c2 |

**Table S6. Branch-Specific Top 20 Genes Sorted by Bayesian Factors.**

| Cardiac | |
| --- | --- |
| CM | EC |
| Tnnc1  Mybpc3  Csrp3  Nkx2-5  Hspb2  Ldb3  Asb2  Cnn1  Cryab  Tnnt2  Slc8a1  Synpo2l  Myom1  Ttn  Actc1  Alpk2  Myh6  Pgam2  Ankrd1  Acta2 | Sox18  Tal1  Icam2  Grap  Flt4  Arhgef15  Cdh5  Pecam1  Myzap  Gngt2  Eng  Adgrf5  Klhl6  Adam15  Zc3hav1  Gypc  Klhl4  Hhex  Igf1  Egfl7 |

**Table S7. Branch-Specific Top 20 Genes Sorted by Bayesian Factors.**

| Haematopoiesis | | |
| --- | --- | --- |
| Eryth | Gran | Mono |
| Mfsd2b  Gata1  Slc14a1  Tal1  Itga2b  Pf4  Sdpr  Klf1  Car1  Gfi1b  Smim5  Gm15915  Apoe  F2r  Zfpm1  Sdsl  Tgfbr3  Ermap  Pus7  Icam4 | Camp  S100a9  S100a8  X1100001G20Rik  Ngp  Pilrb1  Pilrb2  Itgb2l  Lrg1  Abca13  Pglyrp1  Cd177  Fam101b  Cebpe  Prom1  Ncam1  Sept5  Ltb4r1  Ltf  Chil4 | Ly86  Ccr2  Irf8  Pyhin1  Rassf4  Pld4  Csf1r  Ms4a6c  Gria3  Nrp1  Tmem229b  Ass1  BC035044  Ms4a4c  Lgals1  Met  Cx3cr1  Erp29  Milr1 |

S3. Simulating the neuronal differentiation trajectory

Based on a sophisticated system of differential equations encompassing 12 genes, as established (Qiu, et al., 2017), the generation and analysis of simulated data modeled the intricate differentiation processes within the central nervous system. To ensure system stability and maintain predictable dynamics, the nuisance parameter Mature from the original study was deliberately set to zero, thereby eliminating potential chaotic behavior. The regulatory network architecture was structured around two pivotal pairs of mutually inhibitory transcription factors (TFs): Mash1-Hes5 and Scl-Olig2 (Li, et al., 2024; Sagner, et al., 2018). The former orchestrates the bifurcation between neuronal and glial lineages while the latter governs the bifurcation between astrocytic and oligodendrocytic fates. The initial conditions were precisely defined, with all genes initialized to zero except Pax6, which was designated as the sole activated gene to trigger the differentiation cascade.

To solve the stochastic differential equations with high precision, the numerical simulation adopted Euler method. The temporal domain spanned 20 units and discretized into 400 uniform time steps, providing sufficient temporal resolution to capture the subtle dynamics of gene expression patterns. Stochastic effects were incorporated through the systematic introduction of random perturbations at each time step, resulting in nondeterministic differentiation trajectories that accurately reflected the inherent biological variability. To establish statistical robustness, 200 independent simulations were executed, each initiating from an identical progenitor cell state and progressing through distinct differentiation pathways to culminate in one of three terminal cell states: neurons, astrocytes, or oligodendrocytes.

The postsimulation analysis involved a rigorous classification protocol based on the terminal expression levels of the master regulatory genes Mash1, Hes5, Scl, and Olig2 at the final time point (Sagner, et al., 2018). These quantitative expression profiles served as definitive markers for assigning each trajectory to its corresponding terminal cell fate. Furthermore, the temporal progression of cell fate determination was elucidated through detailed analysis of bifurcation points, characterized by the dynamic expression patterns of the transcription factor pairs Mash1-Hes5 and Scl-Olig2, enabling the precise identification of lineage specific differentiation pathways. This methodologically robust simulation framework generated a comprehensive dataset comprising 200 distinct trajectories, effectively capturing the complete developmental progression from progenitor states to terminal cell fates, thereby providing a mathematically rigorous and biologically relevant model of gene regulatory dynamics during cellular differentiation.

S4. Data Preprocessing

Hayashi et al. Data were downloaded from the Gene Expression Omnibus with accession GSE98664. TPM values were used as expression after log_10_（TPM + 1) transformation. Genes were filtered to include those detected in ≥10% of cells and the top 5000 variable transcripts.

Chu et al. Data were downloaded from the Gene Expression Omnibus with accession GSE75748. Raw counts were normalized by the median-of-ratios method and transformed using log₂ (Norm-counts + 1). Genes with very low expression and confounding cell-cycle genes were removed.

Treutlein et al. FPKM values were downloaded from the Gene Expression Omnibus with accession GSE52583. Genes with FPKM ≤ 1 were considered not expressed, and cells with low housekeeping gene expression were excluded. log₂ FPKM values were used for analysis.

Jia et al. Data were downloaded from the ENA (PRJEB23303). Raw counts were normalized using scran deconvolution and transformed into log₂ counts. Genes expressed in fewer than 10 cells or with total count ≤2000 were filtered out.

Olsson et al. Data were downloaded from the Gene Expression Omnibus with accession GSE70245. GSE70245 is merely a superseries stub—the raw expression matrices are found in its four constituent Series: GSE70236 (CMP), GSE70240 (GMP), GSE70243 (LK CD34⁺) and GSE70244 (LSK). For each Series we downloaded the author-provided RSEM expected-counts matrices listed under “processed files,” merged them on Ensembl gene IDs, and initially obtained 394 single-cell libraries (96 LSK, 96 CMP, 136 GMP, 66 LK CD34⁺). We then removed the 12 libraries whose GSM titles include the term “poor quality,” resulting in a final set of 382 high-quality cells used for all downstream analyses. TPM values quantified by RSEM were log₂(transformed and median-centered). Genes expressed in less than ~10% of cells were excluded.

S5. Algorithmic Summaries of Trajectory Inference Methods

**Ouija**
Ouija accepts a small panel of prior marker genes, applies Bayesian nonlinear factor analysis to fit each gene’s expression trajectory, and jointly estimates cell pseudotime within a shared latent space. Through explicit tests of gene-activation dynamics it returns highly interpretable temporal coordinates. The method is sensitive to marker quality and is therefore best used in systems with well-defined regulators and a single dominant differentiation direction.

**Palantir**
Palantir builds a cell-to-cell transition matrix and uses a Markov diffusion process to infer a global pseudotime ordering. It then computes, for every cell, the probability of reaching each terminal fate and employs entropy to quantify developmental plasticity, thereby pinpointing key branch points and regulatory drivers. This probabilistic framework excels in capturing continuous lineage structure and quantifying fate bias.

**Monocle 3**
Monocle 3 partitions cells in a low-dimensional embedding and learns a tree- or cycle-like principal graph through reversed-graph embedding. Pseudotime is defined by cumulative graph distance along this structure, and multiple branches or closed loops can be analysed simultaneously. The method is well suited to large tissue-scale single-cell data sets.

**VIA**
VIA operates on million-cell adjacency graphs by introducing a lazy-teleporting random walk and an augmented diffusion process that captures both global topology and local micro-branches. It reconstructs non-tree or disconnected lineages and delivers high-resolution pseudotime while revealing rare cell fates.

**Slingshot**
Slingshot starts from precomputed clustering and dimensionality reduction, constructs a minimum-spanning tree to delineate lineage branches, fits a smooth principal curve for each branch, and projects individual cells onto these curves to obtain pseudotime. This two-step design combines robustness to noise with flexibility for multiple branching trajectories.

**Diffusion Pseudotime (DPT)**
DPT represents the expression manifold with diffusion maps, selects a root cell, orders all cells by diffusion distance, and uses a Kendall Tau-based heuristic to locate potential branch points. Assuming tree-like diffusion, it remains a standard choice for reconstructing gradual developmental progressions.

**STREAM**
STREAM selects highly variable genes, reduces dimensionality, learns a branching principal tree with ElPiGraph, and retraces cellular evolution along each path. An interactive visual interface and sliding-window analysis provide an intuitive view of transcriptional or epigenomic dynamics over time and space.

**scFates**
scFates builds a principal graph in PCA or diffusion space, chooses a root cell to assign pseudotime, and incorporates statistical tests that highlight branch-specific gene modules. GPU acceleration enables the study of large, highly branched differentiation systems.

**SLICER**
SLICER automatically identifies informative genes, applies locally linear embedding to create a k-nearest-neighbour graph, follows shortest paths to recover pseudotime, and detects branches and loops without supervision. It is suited to complex manifolds where prior markers are absent.

**PAGA**
PAGA abstracts cell clusters into graph nodes, estimates random-walk connectivity to create a topology-preserving coarse graph, computes pseudotime along each inter-cluster path, and uses sliding-window analysis to depict gene-expression trends. The approach scales to data sets containing up to a million cells.

**TSCAN**
TSCAN begins with clustering, constructs a minimum-spanning tree among cluster centroids, projects cells back to this structure to assign pseudotime, and benefits from cluster-level modelling that reduces search complexity and improves noise robustness. A graphical interface supports comparison and quality assessment of alternative orderings.

**Wishbone**
Wishbone targets binary branching trajectories, identifies the main path in diffusion-map space, automatically detects the unique branch point, labels cells to one of the two diverging lineages, and produces a high-resolution timeline. The method is especially useful for cytometry and single-cell RNA-seq data sets exhibiting a clear bifurcation.

SI References

Campbell, K.R. and Yau, C. A descriptive marker gene approach to single-cell pseudotime inference. *Bioinformatics* 2019;35(1):28-35.

Cao, J.*, et al.* The single-cell transcriptional landscape of mammalian organogenesis. *Nature* 2019;566(7745):496-502.

Chen, H.*, et al.* Single-cell trajectories reconstruction, exploration and mapping of omics data with STREAM. *Nature Communications* 2019;10(1):1903.

Chu, C.*, et al.* Routinized Assessment of Suicide Risk in Clinical Practice: An Empirically Informed Update. *Journal of clinical psychology* 2015;71(12):1186-1200.

Faure, L.*, et al.* scFates: a scalable python package for advanced pseudotime and bifurcation analysis from single-cell data. *Bioinformatics* 2022;39(1).

Haghverdi, L.*, et al.* Diffusion pseudotime robustly reconstructs lineage branching. *Nature Methods* 2016;13(10):845-848.

Hayashi, T.*, et al.* Single-cell full-length total RNA sequencing uncovers dynamics of recursive splicing and enhancer RNAs. *Nature Communications* 2018;9(1).

Ji, Z. and Ji, H. TSCAN: Pseudo-time reconstruction and evaluation in single-cell RNA-seq analysis. *Nucleic Acids Research* 2016;44(13):e117-e117.

Jia, G.*, et al.* Single cell RNA-seq and ATAC-seq analysis of cardiac progenitor cell transition states and lineage settlement. *Nature Communications* 2018;9(1).

Li, Z.*, et al.* bHLH transcription factors Hes1, Ascl1 and Oligo2 exhibit different expression patterns in the process of physiological electric fields-induced neuronal differentiation. *Molecular Biology Reports* 2024;51(1):115.

Olsson, A.*, et al.* Single-cell analysis of mixed-lineage states leading to a binary cell fate choice. *Nature* 2016;537(7622):698-702.

Qiu, X.*, et al.* Reversed graph embedding resolves complex single-cell trajectories. *Nature Methods* 2017;14(10):979-982.

Sagner, A.*, et al.* Olig2 and Hes regulatory dynamics during motor neuron differentiation revealed by single cell transcriptomics. *PLOS Biology* 2018;16(2):e2003127.

Setty, M.*, et al.* Characterization of cell fate probabilities in single-cell data with Palantir. *Nature Biotechnology* 2019;37(4):451-460.

Setty, M.*, et al.* Wishbone identifies bifurcating developmental trajectories from single-cell data. *Nature Biotechnology* 2016;34(6):637-645.

Stassen, S.V.*, et al.* Generalized and scalable trajectory inference in single-cell omics data with VIA. *Nature Communications* 2021;12(1):5528.

Street, K.*, et al.* Slingshot: cell lineage and pseudotime inference for single-cell transcriptomics. *BMC Genomics* 2018;19(1):477.

Treutlein, B.*, et al.* Reconstructing lineage hierarchies of the distal lung epithelium using single-cell RNA-seq. *Nature* 2014;509(7500):371-375.

Welch, J.D., Hartemink, A.J. and Prins, J.F. SLICER: inferring branched, nonlinear cellular trajectories from single cell RNA-seq data. *Genome Biology* 2016;17(1):106.

Wolf, F.A.*, et al.* PAGA: graph abstraction reconciles clustering with trajectory inference through a topology preserving map of single cells. *Genome Biology* 2019;20(1):59.
